# Supplementary material for: Decentralising chronic disease management in sub-Saharan Africa: a protocol for the qualitative process evaluation of community-based integrated management of HIV, diabetes and hypertension in Tanzania and Uganda
Source: BMJ Open. 2024 Mar 20;14(3):e078044. doi: 10.1136/bmjopen-2023-078044 (PMC10961519; doi:10.1136/bmjopen-2023-078044)
Supplement: Supplementary data [file bmjopen-2023-078044supp001.pdf]

Supplemental File: Data Collection Guides

Health care users/patients (interview guide)

Introduction

Thank you for consenting to participate in this study. As mentioned to you earlier, this is a collaborative research project involving Ministry of Health, Medical Research Council/Uganda Virus Research Institute/ National Institute for Medical Research-Tanzania and London School of Hygiene and Tropical Medicine Research Unit, University College London and the Liverpool School of Tropical Medicine in UK. The aim of the research is to understand your perspectives as service users towards the management of the chronic disease conditions (HIV, diabetes and hypertension or combination of these) in relation to the integrated care model when health services for these conditions are provided together. Participation is purely voluntary, and refusal does not affect the quality of care that you will receive. We assure you of the confidentiality of the information that you will provide. We request you to allow us to use the tape recorder just to ensure the correctness of the information when writing the report.

Socio-demographic and treatment history

1. Briefly tell me about yourself. *Probe for: age, marital status, education level, source of livelihood, religion, If they have children (how many and ages)*
2. Please tell me about the medical conditions that you are suffering from? *(Probe: diabetes, hypertension, HIV, combination of conditions);*

   -When did you learn that you have this/these conditions?

   -How did you come to know about the condition(s)? *Probe for: Symptoms*

   -What actions did you initially take after learning/knowing that you have this/these conditions? *(Probe for: Self-medication, consultation with health facilities, spiritual healers, took/used herbal medicine prayed etc.)* -And reasons for taking the above action?

   - Whether the options were sought concurrently or simultaneously and why?
3. What do you think are the main causes of the disease that you are suffering from? *Probe for: what are the local/cultural beliefs attached to the cause of the disease(s)?* Probe for information about healthy diet/exercise behaviours
4. How do you think the above-mentioned disease condition can be prevented? *probe for: what are the local/cultural beliefs attached to the cause of the disease(s)?*
5. How do you think patients living with the above mentioned diseases can be better managed?

Perceptions on INTE-COMM

6. What do you know about the integrated community-based care of HTN, DM, and HIV (INTE-COMM)? *Probe for:*

    -Knowledge about INTE-COMM, ability to differentiate INTE-COMM from facility based integration, source of this information, etc.

7. What are your views/perceptions on the integrated community-based care model? *Probe for:*

    -***Willingness/unwillingness*** *of the patients to seek and continue seeking care in such a decentralized integrated model and reasons why*?

    - ***Possible positives*** *about integration e.g. time spent, perceived quality of care, stigma, costs, continuity of care, medicines availability, perceptions of healthcare provider expertise, follow-up support, Interpersonal relationships, etc.)*

    - ***Possible negatives*** *about integration e.g. time spent at the facility, perceived quality of care, stigma, costs, continuity of care, medicines availability, perceptions of healthcare provider expertise, follow-up support, etc.) Probe for childcare responsibilities if applicable*

8. What services do you think should be integrated and delivered with this model? *Probe for: Diagnostic, medication, consultation, health education sessions or all, etc.*) and the reasons why? Probe for maternity services
9. What services do you think should not be integrated and delivered with this model? *Probe for: Diagnostic, medication, consultation, health education sessions or all, etc.*) *and the reasons why?*

Contextual factors

10. Who are the most trusted people by patients in the community to deliver drug refills, screening, and provide health education and where is the focal point and why?
11. Which category of patients are likely to embrace INTE-COMM easily and why? *Probe for: patients in rural vs Urban, educated vs uneducated, men vs women, rich vs poor, young vs aging, those who subscribe to patient clubs' vs none subscribers, etc.*
12. Which categories of patients are likely not to embrace INTE-COMM easily and why? *Probe for: patients in rural vs Urban, educated vs uneducated, men vs women, rich vs poor, young vs aging, those who subscribe to patient clubs' vs none subscribers, etc.*

13. What factors can enable/facilitate patients' easy access to services provided under INTECOMM? *Probe for: Individual, community, and health facility level facilitating factors*
14. What factors make it hard/hinder patients from accessing the services provided under INTE-COMM? *Probe for: Individual, community, and health facility level barriers*. Probe for family responsibilities
15. What do you recommend should be done to enable patients' easy access to services provided under INTE-COMM?
16. What factors do you think enable/facilitate effective/optimal delivery of the community based integrated care model in Uganda? *Probe for: individual-level factors, community level including cultural factors, health systems level, policy level, and political factors, etc.*
17. What factors do you think hinder the effective/optimal delivery of the community-based integrated care model in Uganda? *Probe for*: *individual-level factors, community level including cultural factors, health systems level, and political factors.*
18. What do you recommend should be done to ensure effective delivery of INTE-COMM?
19. Do you think a community-based integrated care model can be sustainable in Uganda? *Probe for*:

    -Factors that can enable the delivery of a sustainable community-based integrated service.

    -Factors that can hinder the delivery of a sustainable community-based integrated service?

Healthcare-seeking experiences of integrated care services

20. Now that you are receiving medical treatment from the health facility/community post, What enables/motivates you to continue seeking health care services for the conditions that you have? *Probe for interpersonal relationships, waiting time, distance to reach the clinic, availability of medicine and supplies, etc.)*
21. What demotivates you in seeking health care services from a health facility/community post for the condition(s) that you have? *Probe for: Interpersonal relationships, waiting time, distance to reach the clinic, availability of medicine and supplies, etc.)* Probe for family responsibilities, childcare, finances
22. What motivates you to comply with the medical treatment provided by the health workers from the health facility/community post for the condition(s) you have?
23. What demotivates you to comply with the medical treatment provided by the health workers from the health facility/community post for the condition(s) you have?

24. For the patients who refused, or dropped out from this intervention(INTE-COOM) what do you think were their fears?
25. How do you suggest should be done to help address these fears in order to have more patients join and benefit from this intervention?

Process factors

26. Kindly tell me about the procedures that you normally follow when you come to receive the services. *Probe for:*

    -How easy is it for you to follow and comply with those stipulated procedures? -How difficult is it for you to follow and comply with those stipulated procedures (probe about stigma, shyness, lack of clarity, etc.)?

    -How do you suggest these procedures should be improved?

27. Tell me about the quality of services that you receive from the health facility/community based integrated care model. *Probe for*: *Interpersonal relationships, waiting time, distance to reach the clinic, availability of medicine and supplies, etc.)*
28. Which services were delivered well and why? *Probe for: health workers' knowledge, competence, availability of medical supplies, the attitude of health workers, inconsistencies in methods of delivery, evidence of patients' outcomes(impact), transport issues, etc.*
29. From the time you joined INTE-COMM, have you noticed any changes in your life? *Probe for: physical changes, economic, psychological, etc.)*
30. Which services were not delivered well and why? *Probe for: health workers' knowledge, competence, availability of medical supplies, the attitude of health workers, inconsistencies in methods of delivery, evidence of patients' outcomes, transport issues etc*
31. What are the recommended best practices for delivering effective community-based integrated care management for HIV, diabetes, and hypertension in Uganda?

Scalability of INTE-COMM

32. What are the factors that can enable/facilitate scaling up a community-based integrated care management of HIV, diabetes, and hypertension in Uganda?
33. What are the factors that can hinder the scaling up of a community-based integrated care management of HIV, diabetes, and hypertension in Uganda?
34. What do you recommend to be done to ensure an effective scaling up of INTE-COMM to other areas in the country? *Probe for: what recommendations, and by whom?*
35. Do you have any other comments in regard to this topic?

Thank you for your participation

Healthcare providers (Interview guide) Introduction

Thank you for consenting to participate in this study. This is a collaborative research project involving Ministry of Health, Medical Research Council/Uganda Virus Research Institute/National Institute for Medical Research-Tanzania and London School of Hygiene and Tropical Medicine Research Unit, University College London and the Liverpool School of Tropical Medicine in UK. In this study we are interested in understanding your reflection towards the health care service integrated model in the management of HV and NCD disease conditions. We have selected some few implementers directly working closely with patients seeking NCD/HIV care at the clinics and the policy makers. You will not be paid for participating in this study but we encourage you to participate because the answers we get from you and several others will be analyzed to get the general picture of the health service delivery integrated model at the community point of care. We are kindly requesting you to take part, and we assure you of the confidentiality of the information that you will provide. We request you to allow me to use the tape recorder just to ensure the correctness of the information when writing the report.

Socio-demographics and chronic disease treatment history

1. First of all, I would like to get the information about you. *Probe for and record: age, marital status, occupation, educational level, title/position, roles in integrated care service delivery*
2. Based on your experience in the management of chronic conditions, what are the main chronic disease conditions that people are suffer from in the community? (*Probe for HIV, diabetes and hypertension*)
3. What are the common health care seeking practices for community members for HIV, DM and HT? and why (*probe on self-medication, consultation with traditional healers, health facilities, spiritual healers)*
4. What services do you offer for these chronic conditions? *Probe for: each condition (DM, HIV, and HTN)*

Perceptions on INTE-COMM

5. What do you know about the integrated community-based care of HTN, DM, and HIV (INTE-COMM)? *Probe for:*

-Knowledge about INTE-COMM, ability to differentiate INTE-COMM from facility-based integration, source of this information, etc.

-Which services are delivered, who delivers the services-diagnostic, medication, consultation, health education sessions or all

-Which services are not integrated and delivered with this model? Probe for: Diagnostic, medication, consultation, health education sessions or all, etc) and the reasons why?

6. What are your views/perceptions on the integrated community-based care model? *Probe for:*

   -***Willingness/unwillingness*** *of the patients to seek and continue seeking care in such a decentralized integrated model and reasons why*?

   - ***Possible positives*** *about integration e.g. time spent, perceived quality of care, stigma, costs, continuity of care, medicines availability, perceptions of healthcare provider expertise, follow-up support, Interpersonal relationships, etc.)*
   - ***Possible negatives*** *about integration e.g. time spent at the facility, perceived quality of care, stigma, costs, continuity of care, medicines availability, perceptions of healthcare provider expertise, follow-up support, etc.)*
7. What services do you think should be integrated and delivered with this model? *Probe for: Diagnostic, medication, consultation, health education sessions or all, etc.*) *and the reasons why?*
8. What services do you think should not be integrated and delivered with this model? *Probe for: Diagnostic, medication, consultation, health education sessions or all, etc.*) *and the reasons why?*
9. What factors can enable patients to accept participating in the INTE-COMM? *Probe for:* ***individual***, ***community,*** *and* ***facility-level*** *enablers to patients' participation*
10. What factors can hinder other patients from accepting to participate in the INTE-COMM? *Probe for:* ***individual***, ***community,*** *and* ***facility-level*** *barriers to patients' participation*
11. What can be done to improve patients' participation in this intervention?

Experience in HIV and NCD management

12. Kindly share with me your experience of delivering INTE-COMM, how satisfied are you with working in the community-based integrated model? *Probe for the reasons why?*
13. What are the patients' attitudes towards community-based integration compared to facility based integration for the management, of HIV, diabetes, and hypertension? *Probe for: evidence of* ***positive*** *and* ***negative*** *attitudes.*
14. What services are provided at the facility/community post for HIV, diabetes and hypertension? ask for one condition at a time. *Probe for: Diagnostic, medication, consultation, health education sessions or all, etc. as per the level of health facility related to HIV, DM, and HT.*

15. At the health facility/community post level, what do you do to ensure HIV, DM, and HT care is user-friendly? *Probe for: patient-health worker relationship, patient-patient relationship, health education, etc.)*
16. Now that you are offering medical treatment from the health facility/community post, What keeps you motivated to continue delivering health care services for your patients with chronic conditions? *Probe for: interpersonal relationships, transportation, allowances, patients' attitudes, physical space, training, patients' response to treatment, availability of medicine and supplies, etc.)*
17. What demotivates you, as you offer health care services to patients with chronic disease from a health facility/community post? *Probe for: interpersonal relationships, transportation, allowances, patients' attitudes, physical space, training, patients' response to treatment, availability of medicine and supplies, etc.)*
18. For the patients who refused, or dropped out from this intervention(INTE-COOM) what do you think were their fears/reasons?
19. What do you suggest should be done to help address these fears in order to have more patients join and benefit from this intervention?
20. While offering health care services for patients with diabetes, hypertension, and HIV under INTE-COMM, what challenges do you face?
21. How can these challenges be addressed to ensure optimal delivery of an effective INTE-COMM?
22. What are the challenges faced by patients in accessing services through INTE-COMM? *Probe for: distance, drug availability, stigma, physical space/infrastructure, time spent, costs, etc.)*
23. How can these challenges be addressed to ensure optimal access to INTE-COMM services?

Process factors

24. Kindly tell me about the procedures that you normally follow to serve your patients when they come to receive the services. *Probe for: -How easy is it for the patients to follow and comply with those stipulated procedures?*

-How difficult is it for patients to follow and comply with those stipulated procedures (probe about stigma, shyness, lack of clarity, etc.)?

-How do you suggest these procedures should be improved?

25. Tell me about the quality of services that you offer to patients through a community-based integrated care model Probe for: Which services were delivered well and why? (*including health workers' knowledge, competence, availability of medical supplies, the attitude of*

health workers, inconsistencies in methods of delivery, evidence of patients' outcomes(impact), transport issues, etc.)

26. Which services were not delivered well and why? *Probe for: health workers' knowledge, competence, availability of medical supplies, the attitude of health workers, inconsistencies in methods of delivery, evidence of patients' outcomes, transport issues, etc*
27. What are the recommended best practices for delivering effective community-based integrated care management for HIV, diabetes, and hypertension in Uganda?
28. What are the factors that may hinder the delivery of effective community-based integrated care services for HIV, DM, and HTN? *Probe for: Individual level, community, or structural/health facility level factors.*
29. . What do you think the best practices are, in delivering effective management of the mentioned chronic disease conditions in the same clinic at the community level?
30. What do you think should be done going forward to enable a robust and sustainable community-based integrated service for HIV/NCD? and by whom?

Scalability of INTE-COMM

31. Based on your experience in health care delivery, what are your views on scaling up INTECOMM to the rest of the areas in the country? Probe for: *-If it is necessary to scale up or not?*
    -How can scaling up of INTE-COM be done/achieved?
32. What are the factors that can enable/facilitate scaling up a community-based integrated care management of HIV, diabetes, and hypertension in Uganda? *Probe for: available strengths and opportunities for scaling up INTE-COMM.*
33. What are the factors that can hinder the scaling up of a community-based integrated care management of HIV, diabetes, and hypertension in Uganda? *Probe for: available weaknesses, and threats to scaling up INTE-COMM.*
34. What do you recommend to be done to ensure an effective scaling up of INTE-COMM to other areas in the country? *Probe for: what recommendations, and by whom?*
35. Do you have any other comments in regard to this topic?

    Thank you for your participation

Policy makers at Ministerial and provincial/regional/district level clinical/health senior management (Director for NCD, HIV and curative services)/ NGO and international organisations (interview guide)

Introduction

Thank you for consenting to participate in this study. This is a collaborative research project involving Ministry of Health, Medical Research Council/Uganda Virus Research Institute National Institute for Medical Research-Tanzania and London School of Hygiene and Tropical Medicine Research Unit, University College London and the Liverpool School of Tropical Medicine in UK. In this study we are interested in understanding your reflection towards the health care service integrated model in the management of HV and NCD disease conditions. We have selected some few implementers directly working closely with patients seeking NCD/HIV care at the clinics and the policy makers. You will not be paid for participating in this study but we encourage you to participate because the answers we get from you and several others will be analysed to get the general picture of the health service delivery integrated model at the community point of care. We are kindly requesting you to take part, and we assure you of the confidentiality of the information that you will provide. We request you to allow me to use the tape recorder just to ensure the correctness of the information when writing the report.

Demographic profile

1. First of all, I would like to get the information about you. *Probe for age, sex, marital status, occupation, educational level, title/position, roles*

Experience of HIV and NCD

2. Based on your experience in the health care system, what are the main chronic disease conditions that people are suffering from? (*Probe for HIV, diabetes and hypertension*).
3. For HIV, diabetes and hypertension, can you describe the burden of disease for each, and if co-/multi-morbid.
4. What can you comment on the care seeking practices for community members for HIV, DM and HT? and why (*probe on self-medication, consultation with traditional healers, health facilities, spiritual healers)*
5. At an institution/organisational level, what does the facility management/district/national do to ensure HIV, DM and HT care is user friendly? *Probe for; diagnostic, drugs availability, health worker availability, waiting area and time, sitting arrangements, health education sessions, encouraging patient-patients' relationship/associations* Probe for:

-The possibility to allocate resources for NCDs management

-What changes have been implemented to address such burden?

Perceptions/Views on INTE-COMM

6. What do you know/have you heard about the integrated model service delivery? what ideas come up in your mind as a leader (*probe for: what it is, who to provide it, expectation-positive and negative*)
7. What services do you think should be integrated? (*Probe: Diagnostic, medication, consultation, health education sessions or all etc*) and the reasons why?
8. What are your perceptions towards such integrated model? (*Probe of the willingness of the patients to continue seeking care in such integrated model, stigma, etc.*)
9. Possible positives and negatives about integration e.g. *time spent at the facility, perceived quality of care, stigma, costs, continuity of care, medicines availability, perceptions of healthcare provider expertise, follow up support etc*.)
10. What are the foreseen factors that can enable successful implementation of a decentralised integrated care model at community level?
11. What are the foreseen factors that can hinder a successful implementation of a decentralised integrated care model at community level?
12. Do you foresee any difficulties in providing an optimal scaling up of community based integrated service?
13. What can be done to help achieve an optimal scaling up of community based integrated services in Uganda?

Thank you for your participation

Community Leaders/Members (Focus group discussion guide) Introduction

Thank you for consenting to participate in this study. As mentioned to you earlier, this is a collaborative research project involving Ministry of Health, Medical Research Council/Uganda Virus Research Institute National Institute for Medical Research-Tanzania and London School of Hygiene and Tropical Medicine Research Unit, University College London and the Liverpool School of Tropical Medicine in UK.

The aim of the research is to understand the Community perspectives towards management of the chronic disease conditions (HIV, diabetes and hypertension or combination of these) in relation to the community based integrated care model. Participation is purely voluntary, and refusal will be treated professionally with no negative repercussion.

Your views will provide the general picture of community care seeking practices including barriers and facilitators, also get opinion on the integrated care service model for patients seeking services for NCD/HIV care at the community health care posts or facility based clinics. You will not be paid for participating in this discussion but we encourage you to participate because the answers we get from you and several others will be analyzed to get the general picture and areas for improvement in strengthening the integrated service delivery model. We assure you of the confidentiality of the information that you will provide. The discussion will last between one to two hours depending on the richness of the information that you have. We request you to allow us to use the tape recorder just to ensure the correctness of the information when writing the report.

Shall we proceed? Yes…….1 No……2

1. May you kindly introduce yourself (age, marital status, residency/ location, and occupation and the position at the village government leadership)

   In our discussion today, we are going to focus on the management of Chronic disease conditions such as HIV, diabetes and hypertension

Community understanding of Chronic Disease Conditions

2. What do you understand by the phrase ‘chronic disease condition’?
3. What are the main chronic conditions people are suffering from in your communities? *(Probe examples; HIV, Diabetes and hypertension).*
4. Based on your experience, what activities or practices in the community that put one at risk of acquiring of HIV, diabetes and hypertension?

5. In your communities, how do people seek health care for HIV, Diabetes and hypertension? Probe on *Self-medication, consulting health facilities, spiritual healers, traditional healers and reasons for such option (probe one after the other for HIV, diabetes and hypertension)*

   Health care services provided for chronic disease conditions (HIV, DM, HT) at the health facilities

6. As leaders, how would you describe the quality of services for people with HIV, DM and HT provided in the nearby health facility? (*Probe for: what do you like/dislike about them? probe trust in existing services)*
   a. Probe on whether they meet the needs of the patients in terms of screening facilities, prescription, availability of medicine, prescribers' *continuity of care, medicines availability, perceptions of healthcare provider expertise*)
   b. What are the key challenges affecting effective management of chronic conditions in your community?
   c. How best can these challenges be addressed to ensure effective management of chronic diseases?

Perceptions on INTE-COMM

7. The Ministry of Health is implementing a study on the integration of health care services for DM/HT/HIV in some selected community posts. This implies that patients with either DM, HT, HIV or both will receive care in a for all the diseases at once from their communities.
   a. What do you know about it (integrated model delivery)? *(probe on whether they have heard from others or is the first time, what ideas come up in your mind as leaders (probe for expectations)?*
   b. What are your views/perceptions towards such integrated model? *(Probe of the willingness of the patients to start and continue seeking care in such integrated model, stigma, and policy issues, cultural factors, etc.)*
   c. What are the possible positives and negatives about integration *e.g. time spent at the facility, perceived quality of care, stigma, costs, follow up support etc.)*

Contextual factors

8. Based on your experience as a leader in your community, what are the foreseen factors that can enable proper implementation of a community based integrated care service model? *Probe for: political support, policy environment, cultural values, beliefs and norms, religious influence, technical factors, etc.*

9. What are the foreseen factors that can hinder the proper implementation of a communitybased integrated care service model? *Probe for: political support, policy environment, cultural values, beliefs and norms, religious influence, technical factors, etc.*
10. What can be done to ensure the effective implementation of a community-based integrated care service model in your community?
11. What role can community leaders play in ensuring effective implementation of a community-based integrated care service model in your community?
12. What are the factors that can hinder the acceptability of INTE-COMM services? *Probe for: political support, policy environment, cultural values, beliefs and norms, religious influence, technical factors, etc*
13. What can be done to ensure the acceptability of a community-based integrated care service model in your community?
14. What role can community leaders play in ensuring acceptability of a community-based integrated care service model in your community?

Sustainability and scalability of INTE-COMM

15. What factors can hinder the sustainable provision of community-based integrated care for HIV, diabetes, and hypertension? *Probe for: political support, policy environment, cultural values, beliefs and norms, religious influence, technical factors, etc*
16. How can community-based integrated care for HIV, diabetes, and hypertension services be sustainably provided in Uganda? *Probe for: what should be done and by who? Including the role of community leaders, etc.*
17. What factors can hinder the scaling up of community-based integrated care for HIV, diabetes, and hypertension? *Probe for: political support, policy environment, cultural values, beliefs and norms, religious influence, technical factors, etc*
18. How can a community based integrated care for HIV, diabetes and hypertension be scaled up to other areas in Uganda? *Probe for the role of different stakeholders*

Thank you for your participation.

Observation Checklist

Instructions:

Observe and document the following in detail

1. Arrival and departure times for both patients and healthcare providers
2. Clinical procedures-how many service points are available, which services are offered at which point, and by whom?
3. Interactions/conversations among patients in the waiting areas, and conversations between patients and health care providers (what issues are talked about)
4. Behaviours of patients-isolating themselves from the rest, patients body language, hunger, restlessness, etc.
5. Behaviours of healthcare providers-language and tone used to communicate with patients, body language/facial expressions, time management, prioritizing patients against other activities, etc.)
6. Sitting arrangements of patients (congestion, comfortable seats, enough seats, exposure to difficult weather conditions, etc.
7. Location of the service points (if offers privacy and confidentiality)
   If health information/education sessions are offered, by whom, what content, participation/attentiveness of patients/asking questions and receiving responses, etc.
