# Supplementary material for: Decentralising chronic disease management in sub-Saharan Africa: a protocol for the qualitative process evaluation of community-based integrated management of HIV, diabetes and hypertension in Tanzania and Uganda
Source: BMJ Open. 2024 Mar 20;14(3):e078044. doi: 10.1136/bmjopen-2023-078044 (PMC10961519; doi:10.1136/bmjopen-2023-078044)
Supplement: Author's manuscript [file bmjopen-2023-078044.draft_revisions.pdf]

BMJ Open

BMJ Open is committed to open peer review. As part of this commitment we make the peer review history of every article we publish publicly available.

When an article is published we post the peer reviewers' comments and the authors' responses online. We also post the versions of the paper that were used during peer review. These are the versions that the peer review comments apply to.

The versions of the paper that follow are the versions that were submitted during the peer review process. They are not the versions of record or the final published versions. They should not be cited or distributed as the published version of this manuscript.

BMJ Open is an open access journal and the full, final, typeset and author-corrected version of record of the manuscript is available on our site with no access controls, subscription charges or pay-per-view fees (http://bmjopen.bmj.com).

If you have any questions on BMJ Open's open peer review process please email info.bmjopen@bmj.com

BMJ Open

Decentralizing chronic disease management in sub-Saharan Africa: a protocol for the qualitative process evaluation of integrated community-based management of HIV, diabetes, and hypertension in Tanzania and Uganda.

Journal:	*BMJ Open*
Manuscript ID	bmjopen-2023-078044
Article Type:	Protocol
Date Submitted by the Author:	22-Jul-2023

Complete List of Authors:	Van Hout, Marie-Claire; Liverpool John Moores University, Public Health Institute<br>Akugizibwe , Mathias; MRC/UVRI and LSHTM Uganda Research Unit<br>Shayo, Elizabeth; National Institute for Medical Research, Policy Analysis and Advocacy<br>Namulundu , Moreen ; MRC/UVRI and LSHTM Uganda Research Unit<br>Kasujja, Francis; MRC/UVRI and LSHTM Uganda Research Unit<br>Namakoola, Ivan; MRC/UVRI and LSHTM Uganda Research Unit<br>Birungi, Josephine; MRC/UVRI and LSHTM Uganda Research Unit,<br>Okebe, Joseph; University College London<br>Murdoch, Jamie; King's College London, School of Life Course and Population Sciences,<br>Mfinanga, Sayoki; National Institute for Medical Research Tanzania, Muhimbili Medical Research Centre; NIMR,<br>Jaffar, Shabbar; University College London, Global Health

Keywords:	DIABETES & ENDOCRINOLOGY, Hypertension < CARDIOLOGY, HIV & AIDS < INFECTIOUS DISEASES

SCHOLARONE™
Manuscripts

Abstract

Introduction: Sub Saharan Africa continues to experience a syndemic of human immunodeficiency virus (HIV) and non-communicable diseases (NCDs). Vertical HIV programming has provided high-quality care for people with HIV in the region, with almost 80% of people living with HIV (PLHIV) in regular care, and 90% virally suppressed. Whilst integrated health education and concurrent management of HIV, hypertension and diabetes is being scaled up in clinics in the region, innovative, more efficient and cost-effective interventions which include decentralisation into the community are required to respond to increased burden of NCDs and co/multi-morbidities.

Methods and analysis: This protocol describes procedures for a qualitative process evaluation of a pragmatic cluster-randomized trial (INTE-COMM) in Tanzania and Uganda which will compare community-integrated care (HIV, diabetes, hypertension) with standard clinic-based integrated care. The model will manage multiple conditions (HIV, hypertension, diabetes) in the community via health monitoring and adherence/lifestyle advice (medicines, diets, exercise) provided by community nurses and trained lay workers, and the devolvement of NCD drug dispensing to the community level. Based on Bronfenbrenner's ecological systems theory, a qualitative process evaluation will be conducted in up to 10 standard care clinics and/or intervention community sites with linked health care facilities. It will include multi-stakeholder interviews (patients, community health workers/volunteers, healthcare providers, policymakers, clinical researchers, international and non-governmental organisation), focus group discussions (community leaders, members) and non-participant observations (community meetings and drug dispensing). These will document and explore various experiences, impacts, dimensions and dynamics from diverse individual and community perspectives at three timepoints in the trial implementation. This synergistic approach will provide findings to explain the main trial findings and offer clear directions for future efforts to sustain and scale up integrated care in Ugandan community settings.

Ethics and dissemination: The protocol has been approved by the University College of London (UK), London School of Hygiene and Tropical Medicine Ethics Committee (UK), Uganda National Council for Science and Technology and the Uganda Virus Research Institute Research and Ethics Committee (Uganda), and the Medical Research Coordinating Committee of the National Institute for Medical

For peer review only - http://bmjopen.bmj.com/site/about/guidelines.xhtml

Research (Tanzania) . University College of London is the trial sponsor. Dissemination of findings will be done through journal publications and stakeholder meetings (study participants, healthcare providers, policy makers and other stakeholders), local and international conferences, policy briefs, peer reviewed journal articles and publications.

Trial registration number ISRCTN15319595. Pre-results.

For peer review only

Strengths and Limitations: Summary Box

The INTE-COMM trial responds to the continued need to tackle the increased burden of NCDs in sub-Saharan Africa, and decentralise healthcare systems into the community to manage co/multi-morbidities with HIV.

A pragmatic cluster-randomized trial will compare community-integrated care for HIV, diabetes and hypertension with standard clinic-based integrated care for the first time in Tanzania and Uganda.

The INTE-COMM trial is based on a partnership between African and UK researchers, working closely with policy-makers, service user representatives, healthcare providers and community members to provide integrated care for PLHIV, diabetes or hypertension in the community.

The process evaluation of INTE-COMM employs qualitative and observational methods at 4-10 facilities to explore and document multi-stakeholder experiences of integration care in the community setting, alongside a broader understanding of the impact of individual, community, familial, social, structural and contextual (community) factors.

The process evaluation operates in tandem with quantitative evaluation of clinical efficacy and cost-effectiveness of the INTE-COMM trial.

Foreseen limitations of the process evaluation may include community site characteristics, lack of service user and community buy-in due to perceptions that community care is inferior to clinic based care, stigma, lack of trust community drug dispensing issues, service user drop-out, social desirability bias and various other contextual mitigating factors.

Key Words

HIV, non-communicable disease (NCD), sub-Saharan Africa, HIV, diabetes, hypertension, decentralisation, community integrated care, Tanzania, Uganda

.

For peer review only

INTRODUCTION

The global burden of non-communicable diseases (NCDs) continues to rise, with most recent data from the World Health Organization (WHO) reporting on an estimated 41 million annual deaths, equivalent to 74% of all deaths globally.[1] NCDs collectively cause 17 million pre-mature deaths in people younger than 70 years per year.[1] Four main NCDs account for over 80% of such premature NCD related deaths.[1] Cardiovascular disease accounts for the majority of NCD related deaths annually (estimated 17.9 million), followed by cancers (9.3 million), chronic respiratory diseases (4.1 million), and diabetes (2 million).[1] Various modifiable lifestyle factors increase the risk of NCD related mortality (e.g. tobacco use, unhealthy diets, physical inactivity, harmful use of substances). According to the 2020 Global Burden of Disease Study the leading metabolic risk factor for NCDs is hypertension (to which 19% of global deaths are attributed) followed by raised blood glucose and overweight/obesity.[2]

NCDs present enormous challenges to progress towards the Sustainable Development Agenda 2030, in terms of reaching targets to reduce reducing the probability of death from any of the four main NCDs between ages of 30 and 70 years by one third by 2030.[1] Mortality and morbidity rates relating to NCDs are concentrated in low-income and middle-income countries (LMICs).[3-6] 77% of all deaths, 86% of premature deaths and 85% of deaths in people aged 30-69 years related to NCDs occur in LMICs.[1] Hypertension and diabetes cause the most NCD related mortality and morbidity in LMICs.[7-8] Women living in LMICs are disproportionately affected by the triple burden of reproductive and maternal health conditions, NCDs, and human immunodeficiency virus (HIV).[4-6] The implementation of poverty reduction initiatives in LMICs are especially hindered by rising HIV/NCD multi-morbidities due to increased household costs related to healthcare, and higher mortality and morbidity of the vulnerable and socially disadvantaged.[1]

The sub-Saharan African (SSA) region continues to experience a syndemic of HIV and NCDs.[1,9--14] The region accounts for 55% of the total 38 million people living with HIV (PLHIV) globally.[15] In addition to the rise in numbers in regular HIV care (almost 80%) and virally suppressed (90%),[16] NCDs among PLHIV in SSA are also increasing due to various HIV related reasons (HIV infection itself, anti-retroviral treatment (ART)) and from life course or aging related risks of chronic co-morbidities (diabetes, hypertension, cancers, metabolic disorders).[17-20] Key relevant socio-economic

For peer review only - http://bmjopen.bmj.com/site/about/guidelines.xhtml

factors fuelling the NCDs rise in the region are increased urbanisation, poor lifestyle and poverty, NCD related healthcare costs (for example drugs) and with vulnerable and poor populations substantially impacted by chronic illness.[9,11-12,21]

Prevention, screening, treatment and care of NCDs (including palliative care) are key components of the NCD response.[1] Long term management of diabetes and hypertension among PLHIV remains a major health system challenge in SSA[22-23] where earlier age of onset of disease of diabetes and hypertension is increasingly observed, and with rising rates of HIV multi-morbidity.[11,12, 14, 24-27] In response to this, efforts to coordinate NCD programmes with scaled up NCD drug supply alongside with the high quality care provided by vertical HIV programming have intensified.[28] There is a growing evidence base and shift toward the implementation of integrated NCD/HIV care provision ('*the coordination, co-location or simultaneous delivery of communicable and non-communicable services to patients'*) in various SSA countries (e.g. in Malawi, South Africa, Botswana, Uganda, Kenya, Tanzania).[22, 29-41] Various systematic and scoping reviews, Delphi consensus studies and implementation projects in SSA indicate that integration of HIV/diabetes and hypertension management can reduce duplication and fragmentation of services by reorganising clinics and staff; streamline services by detecting and treating those with co/multi-morbidities; support an effective, quality and sustainable drug supply chain; increase viral suppression rates and achieve better control of blood pressure/blood glucose; incur service user benefits relating to time investment attending appointments and cost; support uptake, retention and adherence to treatment; encourage greater sharing of health literacy and lay knowledge between service users and providers, and reduce HIV related stigma.[21, 42-57] See **Figure 1.**

Insert **Figure 1: Benefits of integrated HIV, hypertension and diabetes care in general** about here

Community care exists in many forms across the SAA region. Community models for chronic NCD conditions are however rare.[58-59] In contrast, the differentiated community or home based care of HIV using outreach, lay health workers and peer support models is expanding in various SSA countries (Uganda, Mozambique, Kenya).[60-62] Whilst integrated health education and concurrent management of HIV, hypertension and diabetes is being scaled up in clinics in the region, innovative, more efficient and cost-effective interventions which include decentralisation into the community are required to

For peer review only - http://bmjopen.bmj.com/site/about/guidelines.xhtml

respond to increased burden of NCDs and co/multi-morbidities.

To the best of our knowledge there has been no clear evidence to date for a community-based model of integrated HIV& NCD care in SSA. Our Research Partnership for the Control of Chronic Diseases in Africa (RESPOND-AFRICA) partnership[i] will respond to this need to generate evidence which can be used to inform and support decentralisation of healthcare systems to community level in the region to manage NCD related co/multi-morbidities with HIV. We present here the qualitative process evaluation protocol which will compare community-integrated care (HIV, diabetes, hypertension) with standard facility-based integrated care (protocol number ISRCTN15319595).

The INTE-COMM Project

RESPOND-AFRICA has operated in Tanzania and Uganda for many years. We have piloted integrated HIV/NCD care in a feasibility single arm intervention study in selected facilities in the Wakiso district in central Uganda, and Dar-es-salaam and Coastal regions in Tanzania in the Management of Chronic Conditions in Africa (MOCCA) project (2018-2020)[ii],[21, 29, 38, 41] and scaled up integrated care for HIV, diabetes and hypertension at selected facilities in a pragmatic parallel arm cluster randomised trial in the Integrating and decentralising HIV, diabetes and hypertension services in Africa (INTE-AFRICA trial) (2019-2023)[iii]. [39-40, 63-64]

INTE-COMM is a 4-year research project funded by the National Institute for Health and Care Research (NIHR). We will closely adhere to the Medical Research Council (MRC) framework for developing and evaluating complex interventions and frameworks for intervention adaptation and multimorbidity interventions. In a formative phase of 18 months, we have been guided by community-based models of HIV care that include provision of medicines, peer-support and self-management, and a series of collaborative stakeholder consultations with policy makers and patient (service user) groups to design a new community-based integrated model of care for the management of patients with HIV, diabetes and hypertension. This has informed the design of a pragmatic cluster-randomized trial which will compare a community-integrated care model with standard clinic-based integrated care in Tanzania and Uganda. The model will differ from existing community care approaches by managing multiple conditions (HIV, hypertension, diabetes) in the community via health monitoring and adherence/lifestyle advice (medicines, diets, exercise) provided by community nurses and trained lay

For peer review only - http://bmjopen.bmj.com/site/about/guidelines.xhtml

workers, and the devolvement of NCD drug dispensing to the community level. Whilst the project is situated in Tanzania and Uganda, we intend to generate evidence potentially transferable to other community settings in SSA and wider continent.

Patients with HIV, diabetes, or hypertension who are considered stable on treatment at the health facilities will be invited to join the trial. The trial will evaluate whether integrated community care will be effective in improving patient outcomes among PLHIV, diabetes, and hypertension as compared to standard clinic-based integrated care (and including outcomes regarding uptake, retention, acceptability, costs, linkage to clinic-based services and potential cost-effectiveness of integrated community care). Patients living with HIV, diabetes, or hypertension, stable on treatment at clinics, will be organized into groups of approximately 8-14 persons and randomized to integrated community or integrated clinic-based care. The study has two co-primary endpoints; a composite endpoint of glycemia and blood pressure control among individuals with diabetes and/or hypertension and plasma viral load suppression among PLHIV. In the integrated community arm, participants will receive their drugs, adherence support, and monitoring at a community venue. Clinic based care will comprise of integrated health education and clinical management of HIV, diabetes and hypertension. See **Figure 2.**

Insert **Figure 2. INTE-COMM study schema** about here

All study participants will be followed up for 12 months. A sample size of 116 groups will provide over 80% power to detect an absolute difference in blood pressure and blood glucose control of 10% at the 5% two-sided significance level. For HIV viral suppression, the trial will have over 80% power to show non-inferiority with a margin of delta= 8.5%, 7.5%, and 5.5% assuming viral suppression is 85%, 90%, and 95% respectively. To allow for losses to follow-up, our target for enrolment is 124 groups each comprising 14 participants.

Qualitative Process Evaluation of the INTE-COMM trial

Process evaluations typically evaluate how and whether interventions are delivered as intended and whether such implementation is congruent with the theory underpinning the intervention.[65-69] The MRC guidance for evaluation of complex health interventions underscores the importance of process evaluation within trials.[66] Building on experiences and lessons learnt in our process evaluation of the INTE-AFRICA trial,[63] the process evaluation of INTE-COMM will focus explicitly on the various

For peer review only - http://bmjopen.bmj.com/site/about/guidelines.xhtml

system dimensions pertinent to the community care context; e.g. the description of the intervention and its causal assumptions; levels of disease related help seeking behaviours in the community; implementation processes; blocks to uptake and challenges in the community; mechanisms of service user/ provider and community outcomes.

Using qualitative and observational approaches we will document and explore the experiences, attitudes and practices of a wide variety of stakeholders during implementation in order to develop a process-led understanding of the impact of broader social, structural and contextual factors on initiation, delivery and implementation of community-based HIV/NCD service integration over time. We will also assess fidelity, clarify causal mechanisms, identify contextual factors associated with variation in outcomes between community and clinic based integrated care, and assess to what extent resources and activities are supporting the INTE-COMM trial in delivering intended outputs and related clinical and health economics outcomes. A central focus lies in identifying challenges and contextually relevant strategies or solutions in the community setting which can support and guide successful implementation and sustainability of community based integrated prevention, detection and management of HIV with hypertension and diabetes. We are cognisant that this community approach is not devoid of controversy as service users will be moved away from traditional clinic-based care where they were managed by certified clinicians to a community venue where services are delivered by a trained lay worker supported by a nurse. Patients can perceive this as inferior and a far cry from those managed in standalone specialist clinics. We also appreciate that HIV is a stigmatising condition and putting patients together with others without the condition in a community setting may be disconcerting. These potential limitations to effective delivery of the intervention will be carefully monitored within this process evaluation, and insights used to both explain trial findings and inform future wide-scale implementation.

Theoretical framework: Ecological systems theory

The process evaluation will adapt Bronfenbrenner's ecological systems theory [70-71] to conceptualise the INTE-COMM intervention as an event which *'disrupts'* complex social systems operating across multiple contextual levels.[67, 72] The theory provides an organising structure to facilitate in-depth understanding of how implementation of the intervention is shaped by the wider context, and importantly for this study, it's interaction with the local communities into which INTE-COMM is

For peer review only - http://bmjopen.bmj.com/site/about/guidelines.xhtml

introduced.[63,67, 70-73] Drawing on our previous primary care research in LMIC settings,[73] three elements of context, (see Figure 3), will be investigated to capture variation in adoption, delivery and maintenance as well as responses to this community based integrated care intervention, affecting both reach and fidelity, which are likely to be important factors in outcome differences. See **Figure 3.**

Insert **Figure 3: Potential contextual influences on INTE-COMM programme implementation cascade** about here

METHODS AND ANALYSIS

Study setting

Tanzania and Uganda were chosen as study sites given their pioneering work in this area.[29,38-40,62-63,74-75] Their public health services are strongly committed to providing services for NCDs, and are struggling to scale-up provision for diabetes and hypertension in the face of competing health demands, including from HIV and diabetes and hypertension across all socioeconomic strata.[74] See **Table 1**.

Insert **Table 1: Profile of Tanzania and Uganda regarding prevalence of three chronic conditions (hypertension, diabetes, and HIV)** about here

Study design

This protocol describes procedures for a qualitative process evaluation of a pragmatic cluster-randomized trial which will compare community-integrated care with standard facility-based integrated care in Tanzania and Uganda. The process evaluation (interviews, focus groups and observation) works in tandem with the collection of clinical outcomes and health economic data to estimate the potential benefits to patients and health services at community and clinic level. Methods will include multi-stakeholder interviews (patients, community health workers/volunteers, healthcare providers, policymakers, clinical researchers, international and non-governmental organisation), focus group discussions (community leaders, members) and clinic observations. These will document and explore various experiences, impacts, dimensions and dynamics from diverse individual and community perspectives at three timepoints in the trial implementation.

This design permits an assessment of theoretical fidelity of INTE-COMM's implementation, understood for how the intervention is flexibly adapted to function within localised community contexts, rather than a strict assessment of fidelity.[67-68] To do so requires a detailed description of the

For peer review only - http://bmjopen.bmj.com/site/about/guidelines.xhtml

processes, relationships, and contexts involved in the delivery of community based integrated care, and the identification of factors attributing to the failure or success of the programme. It addresses the *'black box'* problem in interpreting trial results by improving understanding of the mechanisms that connect particular intervention components to particular outcomes.[76] The chosen approach will enhance social construction and acceptability of community level integrated approaches, link outcomes to policy and advocacy, improve linkage of surveillance and care between community and clinics, and potential sustainability of HIV, diabetes and hypertension chronic disease service integration in Tanzania and Uganda. This synergistic approach to evaluate, understand and respond will support refinement of community-integrated care (HIV, diabetes, hypertension), inform community and clinic surveillance, drug dispensing development, community nurses and lay workers' practice development and policy reforms to involve and educate communities, and future efforts to sustain and scale up in Tanzanian and Ugandan community settings. See **Table 2**.

Insert **Table 2 Objectives, contextual features and data collection methods of INTE-COMM process evaluation** about here

Study population and recruitment

Data collection involving qualitative and observational techniques will be undertaken at 4-10 representative community and clinic sites (rural, urban/semi urban) and at three timepoints in trial implementation, at the baseline, at six months, and at 12 months. A purposive sampling approach will recruit participants (See **Table 2)**. Service users recruited from those who have already consented to participate in the main trial from both arms, of both genders and on stable treatment for either hypertension, diabetes, HIV, or multi-morbidity will be recruited for face-to-face interview (IDI) with the help of gatekeepers (clinic and community nurses). Replacement of participants (e.g. service users) will occur if significant loss to follow-up or refusal of repeated interviews occurs. Healthcare service providers at both arms (health facility managers, physicians/clinicians, nurses, lay workers, patient club facilitators/peer educators and traditional healers) will also be invited for face-to-face interview (IDIs). Key ministerial policymakers and provincial/regional/district level clinical/health senior management (Director/programme manager/Commissioners, Assistant Commissioner of NCDs; Programme manager/ Commissioner of the National AIDS control program and others), non-governmental and

For peer review only - http://bmjopen.bmj.com/site/about/guidelines.xhtml

international organisations representatives will be identified based on the INTE-AFRICA listing and will be invited for face to face or telephonic interview where it is difficult to meet physically (KII). Community leaders and members will be identified by gatekeepers in the clinic catchment areas and invited to partake in FGDs. Clinical researchers in the INTE-COMM trial will be interviewed and asked to provide reflections on the implementation process (see **Table 2**).

Data collection

Data collection will explore multi-stakeholder perceptions and experiences of the decentralized integrated management of HIV, diabetes, and hypertension care in community and clinic base settings. Interview and focus group guides will be designed based on a scoping review of literature and process evaluation team consultation with the trial implementers. The aim is to develop a phenomenological understanding[77] of the experiences, processes and contextual factors that influence the implementation of the intervention within community eco-and cultural systems. Factors that can potentially influence the sustaining and scaling up of integrated management of HIV, diabetes, and hypertension in the community and at clinic levels will be identified and explored. We include an explicit objective to monitor and identify potential sources of contamination and dilution of the intervention, for example whether other communities are adopting a similar approach to the integrated community intervention or whether people in intervention communities are going elsewhere.

Qualitative investigations will continue until data saturation is reached at each time point. Where possible, we will gender-match the interviewer with participant, particularly in the case of service users. In Tanzania, all interviews and focus groups will be conducted in Swahili language, in Uganda interviews with service users and focus groups with community members/leaders of medicine clubs will be conducted in local languages and the remainder will be conducted in English. Data collection tools will therefore be translated accordingly. Audio recordings will be taken with written and verbal consent and transcribed verbatim by the team. Back translation of transcripts from the local language into English will occur for consistency.

During clinical observations, structured observations supported by field notes will be taken to provide a description of the processes followed and the content involved in delivering integrated care.

For peer review only - http://bmjopen.bmj.com/site/about/guidelines.xhtml

Data analysis and synthesis

Using an approach developed in our previous health systems research [63, 73] we will adopt an ethnographic approach to analyse both theoretical fidelity and the interaction between the INTE-COMM intervention, local communities and the wider health system context. This will be achieved by: 1) setting out relevant ***macro*** (e.g. cultural discourses of NCDs); ***meso*** (e.g. protocols used in monthly meetings, structural arrangements at service points to observe privacy and confidentiality, lay health worker/nurse arrangements); and ***micro*** (e.g. talk and behaviour at dispensaries) contextual features relevant to implementation; 2) targeting where likely tensions in implementation are likely to occur at each contextual level; then 3) analysing tensions within targeted activities involved in intervention delivery; and 4) considering the consequences of these tensions for how the intervention was implemented and the implications of these for scaled up implementation. Tensions in implementation are likely to be manifested in training sessions, interactions between lay health workers, peer educators, nurses and members of the community, or through organisational processes for ensuring the intervention is delivered.

To manage the quantity and range of data collected as part of the process evaluation, analysis will involve working laterally across data types and situating evidence within the ***macro, meso, micro*** contextual framework. We will seek to provide a broad description of intervention delivery but, instead of allocating equal time to the analysis of each case, we will focus on identifying '*telling cases',* triangulating and looking for connections between data.[78] The analysis of qualitative data will be iterative, moving between data collection points and data analysis to test emerging theories. Care will be taken to identify and follow up deviant cases which do not fit into emerging theories. Emerging theories and the relationship of the data to the conceptual literature underpinning the intervention will be discussed and refined at team meetings throughout the research. Inductive analysis will further unpack how service users, providers, other stakeholders and their communities understand and apply diverse lay health literacies, autonomies and decision-making processes including health care seeking regarding the three conditions of interest (HIV, diabetes and hypertension) and settings of clinical care.[63,67, 76] An electronic data management package (NVivo.14) will support the process.

For peer review only - http://bmjopen.bmj.com/site/about/guidelines.xhtml

Credibility and transferability

The process evaluation protocol adheres to recommendations intended to facilitate the standardisation of process evaluation design and reporting.[67] The credibility and reliability of our data will be anchored in the ability to triangulate socio-behavioural and observational data during analysis and CFIR framework mapping in order to better understand how different types of evidence enhance overall interpretation of community based integrated care. Triangulation will occur across theories, methods (qualitative, observations) between countries (Tanzania and Uganda) and sources of data (narratives, checklists, notes).

The process evaluation also operates concurrently with clinical outcomes data and health economics analysis. Taken collectively process evaluation data with clinical and health economics data can inform scalability and transferability to other community settings.

We recognise the potential for selection and information bias as limitations of the trial itself, and mitigate by using a standardised approach to collecting data with continual assessment of information bias, and ensuring that research personnel are unaware of participant disease status. Social desirability is addressed in the process evaluation by only providing brief information at the outset of the evaluation in order to avoid priming, using an interview schedule approved by a panel of INTE-COMM experts in terms of sensitivity, conducting qualitative research using skilled local interviewers with limited power relationship between interviewer and participant, conducting the interviews in a safe and secure setting where the participant feels comfortable or using technology/telephone, briefing them that there is no right and wrong answer, and finally by encouraging them to use anecdotes and experiential evidence to support their views.

Patient and public involvement

As with all RESPOND-AFRICA projects, proactive community engagement is crucial for the success of INTE-COMM. The INTE-COMM trial is cognisant of the importance of public and patient involvement (PPI) throughout its programme of research, and values PPI in enhancing research quality and relevance by providing different perspectives and a sense of ownership. The process evaluation will allow multi-stakeholder voices to be heard, and utilised. Key community level stakeholders such as service users and their families, community members and community care professionals will be fully

For peer review only - http://bmjopen.bmj.com/site/about/guidelines.xhtml

involved in guiding the research, acting as research participants, and in implementation of change in community level integrated health service delivery and integrated care planning. All aspects of the process evaluation are underpinned by participatory action health research and its success and usefulness will be grounded in PPI, participation and engagement in the form of patient/professional identification of research priorities, collaborations and partnerships, expert steering, community participation around health needs and optimal integrated services, awareness raising activities, development of print materials, toolkits and training for community nurses, lay members and healthcare professionals.

ETHICS AND DISSEMINATION

The protocol has been approved by the University College of London (UK), London School of Hygiene and Tropical Medicine Ethics Committee (UK), Uganda National Council for Science and Technology and the Uganda Virus Research Institute Research and Ethics Committee (Uganda) and from the Medical Research Coordinating Committee of the National Institute for Medical Research (Tanzania). University College of London is the trial sponsor. The key ethical principles of voluntary and informed participation, respect of persons, do no harm, autonomy, privacy, anonymity, confidentiality, and safety of participants will be used in all researcher and participant interactions. Written informed consent for participation in interviews and focus groups will be obtained from all participants. All participants will be provided with written information about the research, this will be explained verbally, and informed that their participation is voluntary and that they may withdraw from participation at any time without any penalty. Safety of all data will be ensured by: (1) all transcripts will be encrypted password-protected protected code; (2) data will be uploaded to an encrypted database that will only be accessible by the INTE-COMM research team; (3) all personal identifying information of participants will be kept separately from all transcripts and each participant given a unique participant identification number to delink the information from the personal identifiers; (4) on completion of all the study activities, all audio recordings will be destroyed. The transcript files will be uploaded onto the encrypted database where they will be stored for up to 10 years and thereafter, a decision on how to destroy them will be reached by the study team and partners.

For peer review only - http://bmjopen.bmj.com/site/about/guidelines.xhtml

Timely communication and publication of findings across a broad range of audiences is essential to drive policy considerations around community-based integrated HIV/NCD care in Africa. Through targeted dissemination, communication, outreach and provision of innovative tools and models, the project's long-term conceptual, instrumental, capacity building and network influences will centre on achieving an enhanced understanding of the intricacies involved in integrating HIV and NCD service and patient care at the community level. Dissemination and communication will fully and regularly utilise all established and innovative channels (for example journal publications, conferences, press releases, webinars) target all stakeholders, including a variety of audiences and in local languages and English.

Authors Contributions

All authors contributed to the conceptualisation of the research and contributed to writing the manuscript.

MCVH, MA, EHS, MN, IN, JM, AK, GSM, SJ designed the process evaluation protocol.

SJ, JO, FK, JB led the development of the INTE-COMM trial.

MCVH drafted the manuscript and all co-authors edited and commented on subsequent drafts. All authors approved the final draft for submission. All authors agree to be accountable for all aspects of the work in ensuring that questions related to the accuracy or integrity of any part of the work are appropriately investigated and resolved.

Funding Acknowledgement:

'This work was supported by NIHR; GHPSR Project: NIHR 131273

Competing interests: None. Authors have no conflict of interest to declare.

Patient consent: Not required.

Ethics approval: The protocol has been approved by the University College of London (UK), London School of Hygiene and Tropical Medicine Ethics Committee (UK), Uganda National Council for Science and Technology and the Uganda Virus Research Institute Research and Ethics Committee (Uganda), and the Medical Research Coordinating Committee of the National Institute for Medical Research (Tanzania).

For peer review only - http://bmjopen.bmj.com/site/about/guidelines.xhtml

For peer review only

Provenance and peer review: Not commissioned; internally peer reviewed.

Data sharing statement: No additional data are available.

Open access: This is an open access article distributed in accordance with the Creative Commons Attribution 4.0 Unported (CC BY 4.0) license, which permits others to copy, redistribute, remix, transform and build upon this work for any purpose, provided the original work is properly cited, a link to the licence is given, and indication of whether changes were made. See: https:// creative commons. org/ licenses/ by/ 4. 0/.

REFERENCES

1. World Health Organization (WHO). *Noncommunicable diseases.* World Health Organization, 2022. Available at www.who.int/news-room/fact-sheets/detail/noncommunicable-diseases (Accessed 16 February 2023)
2. Global Burden of Disease Collaborative Network. *Global Burden of Disease Study 2019* Available at https://vizhub.healthdata.org/gbd-results/
3. Das, M. WHO urges immediate action to tackle non-communicable diseases. *Lancet Oncology,* 2022. Early Online. DOI:https://doi.org/10.1016/S1470-2045(22)00606-4
4. Alwan A. *Global status report on noncommunicable diseases 2010.* World Health Organization, 2011.
5. Hoffman RM, Newhouse C, Chu B, Stringer JS, Currier JS. Non-communicable Diseases in Pregnant and Postpartum Women Living with HIV: Implications for Health Throughout the Life Course. *Current HIV/AIDS Reports* 2021, 18(1):73-86.
6. NCD Alliance. *A call to action: women and non-communicable diseases.* 2022. Available at Women's Health | NCD Alliance (Accessed 16 February 2023)
7. Adler AJ, Prabhakaran D, Bovet P, Kazi DS, Mancia G, Mungal-Singh V, Poulter N. Reducing cardiovascular mortality through prevention and management of raised blood pressure. *Global Heart* 2015, 10(2):111-122.
8. Bennett JE, Kontis V, Mathers CD, Guillot M, Rehm J, Chalkidou K, Kengne AP, Carrillo-Larco RM, Bawah AA, Dain K.NCD Countdown 2030: pathways to achieving Sustainable Development Goal target 3.4. *The Lancet* 2020, 396(10255):918-934.

For peer review only - http://bmjopen.bmj.com/site/about/guidelines.xhtml

9. Addo J, Smeeth L, Leon DA: Hypertension in sub-saharan Africa: a systematic review. *Hypertension* 2007;50:1012–8.

10. Remais JV, Zeng G, Li G, Tian L, Engelgau MM. Convergence of non-communicable and infectious diseases in low-and middle-income countries. *International Journal of Epidemiology* 2013, 42(1):221-227.

11. Chang AY, Gómez-Olivé FX, Manne-Goehler J, Wade AN, Tollman S, Gaziano TA, Salomon JA. Multimorbidity and care for hypertension, diabetes and HIV among older adults in rural South Africa. *Bulletin of the World Health Organization* 2019, 97(1):10.

12. George S, McGrath N, Oni T. The association between a detectable HIV viral load and non-communicable diseases comorbidity in HIV positive adults on antiretroviral therapy in Western Cape, South Africa. *BMC Infectious Diseases* 2019, 19(1):1-11.

13. Patel P, Rose CE, Collins PY, *et al*. Noncommunicable diseases among HIV-infected persons in low-income and middle-income countries: a systematic review and meta-analysis. *AIDS* 2018;32 Suppl 1:S5–20.

14. Manne-Goehler J, Atun R, Stokes A, *et al*. Diabetes diagnosis and care in sub-Saharan Africa: pooled analysis of individual data from 12 countries. *Lancet Diabetes Endocrinology* 2016;4:903–12.

15. UNAIDS. *Fact Sheet- World AIDS Day 2021*. Available at: https://www.unaids.org/en/resources/fact-sheet (Accessed 16 February 2023)

16. Ford N, Ball A, Baggaley R, Vitoria M, Low-Beer D, Penazzato M, Vojnov L, Bertagnolio S, Habiyambere V, Doherty M. The WHO public health approach to HIV treatment and care: looking back and looking ahead. *The Lancet Infectious Diseases* 2018, 18(3):e76-e86.

17. Ciccacci F, Tolno VT, Doro Altan AM, Liotta G, Orlando S, Mancinelli S, Palombi L, Marazzi MC. Noncommunicable Diseases Burden and Risk Factors in a Cohort of HIV+ Elderly Patients in Malawi. *AIDS Research and Human Retroviruses.* 2019 35(11-12):1106-1111.

18. Achwoka D, Oyugi JO, Mutave R, Munywoki P, Achia T, Akolo M, Muriuki F, Muthui M, Kimani J: High prevalence of non-communicable diseases among key populations enrolled at a large HIV prevention & treatment program in Kenya. *PLoS One* 2020, 15(7):e0235606.

For peer review only - http://bmjopen.bmj.com/site/about/guidelines.xhtml

19. Achwoka D, Waruru A, Chen TH, Masamaro K, Ngugi E, Kimani M, Mukui I, Oyugi JO, Mutave R, Achia T *et al*: Noncommunicable disease burden among HIV patients in care: a national retrospective longitudinal analysis of HIV-treatment outcomes in Kenya, 2003-2013. *BMC Public Health* 2019, 19(1):372.
20. Kamkuemah, M., Gausi, B. & Oni, T. Missed opportunities for NCD multimorbidity prevention in adolescents and youth living with HIV in urban South Africa. *BMC Public Health* 2020; 20, 821.
21. Shiri T, Birungi J, Garrib AV, Kivuyo SL, Namakoola I, Mghamba J, Musinguzi J, Kimaro G, Mutungi G, Nyirenda MJ. Patient and health provider costs of integrated HIV, diabetes and hypertension ambulatory health services in low-income settings—an empirical socio-economic cohort study in Tanzania and Uganda. *BMC Medicine* 2021, 19(1):1-15.
22. Jaffar S, Ramaiya K, Karekezi C, Sewankambo N, Katahoire AR, Kraef C, Mutungi G, Musinguzi J, Birungi J, Garrib A. Controlling diabetes and hypertension in sub-Saharan Africa: lessons from HIV programmes. *The Lancet* 2021, 398(10306):1111-1113.
23. Haregu TN, Setswe G, Elliott J, Oldenburg B. Integration of HIV/AIDS and noncommunicable diseases in developing countries: rationale, policies and models. *International Journal of Healthcare* 2015, 1(1):21.
24. Guwatudde D, Nankya-Mutyoba J, Kalyesubula R, Laurence C, Adebamowo C, Ajayi I, Bajunirwe F, Njelekela M, Chiwanga FS, Reid T. The burden of hypertension in sub-Saharan Africa: a four-country cross sectional study. *BMC Public Health* 2015, 15(1):1-8.
25. Jaffar S, Gill G. The crisis of diabetes in sub-Saharan Africa. *Lancet Diabetes and Endocrinology* 2017, 5(8):574-575.
26. Jaffar S. Diabetes and other non-communicable diseases in Africa: a potential disaster in the waiting. *Lancet Diabetes & Endocrinology* 2016, 4(11):875-877.
27. Price AJ, Crampin AC, Amberbir A, Kayuni-Chihana N, Musicha C, Tafatatha T, Branson K, Lawlor DA, Mwaiyeghele E, Nkhwazi L. Prevalence of obesity, hypertension, and diabetes, and cascade of care in sub-Saharan Africa: a cross-sectional, population-based study in rural and urban Malawi. *Lancet Diabetes & Endocrinology* 2018, 6(3):208-222.

For peer review only - http://bmjopen.bmj.com/site/about/guidelines.xhtml

28. Ameh S, Klipstein-Grobusch K, D'ambruoso L, Kahn K, Tollman SM, Gómez-Olivé FX. Quality of integrated chronic disease care in rural South Africa: user and provider perspectives. *Health Policy and Planning* 2017, 32(2):257-266.
29. Birungi J, Kivuyo S, Garrib A, Mugenyi L, Mutungi G, Namakoola I, Mghamba J, Ramaiya K, Wang D, Maongezi S. Integrating health services for HIV infection, diabetes and hypertension in sub-Saharan Africa: a cohort study. *BMJ Open* 2021, 11(11):e053412.
30. Divala OH, Amberbir A, Ismail Z, Beyene T, Garone D, Pfaff C, Singano V, Akello H, Joshua M, Nyirenda MJ. The burden of hypertension, diabetes mellitus, and cardiovascular risk factors among adult Malawians in HIV care: consequences for integrated services. *BMC Public Health* 2016, 16(1):1-11.
31. Duffy M, Ojikutu B, Andrian S, Sohng E, Minior T, Hirschhorn LR. Non-communicable diseases and HIV care and treatment: models of integrated service delivery. *Tropical Medicine & International Health* 2017, 22(8):926-937.
32. Golovaty I, Sharma M, Van Heerden A, van Rooyen H, Baeten JM, Celum C, Barnabas RV. Cost of integrating non-communicable disease screening into home-based HIV testing and counseling in South Africa. *Journal of Acquired Immune Deficiency Syndromes* 2018, 78(5):522.
33. Iwelunmor J, Ezechi O, Obiezu-Umeh C, Gbajabiamila T, Musa AZ, Oladele D, Idigbe I, Ohihoin A, Gyamfi J, Aifah A. Capabilities, opportunities and motivations for integrating evidence-based strategy for hypertension control into HIV clinics in Southwest Nigeria. *PloS One* 2019, 14(6):e0217703.
34. Fairall LR, Folb N, Timmerman V, Lombard C, Steyn K, Bachmann MO, Bateman ED, Lund C, Cornick R, Faris G. Educational outreach with an integrated clinical tool for nurse-led non-communicable chronic disease management in primary care in South Africa: a pragmatic cluster randomised controlled trial. *PLoS Medicine* 2016, 13(11):e1002178.
35. Kwarisiima D, Atukunda M, Owaraganise A, Chamie G, Clark T, Kabami J, Jain V, Byonanebye D, Mwangwa F, Balzer LB. Hypertension control in integrated HIV and chronic disease clinics in Uganda in the SEARCH study. *BMC Public Health* 2019, 19(1):1-10.

For peer review only - http://bmjopen.bmj.com/site/about/guidelines.xhtml

36. Masupe T, Mashalla Y, Seloilwe E, Jibril H, Medhin H. Integrated management of HIV/NCDs: knowledge, attitudes, and practices of health care workers in Gaborone, Botswana. *African Health Sciences* 2019, 19(3):2312-2323.

37. Some D, Edwards JK, Reid T, Van den Bergh R, Kosgei RJ, Wilkinson E, Baruani B, Kizito W, Khabala K, Shah S. Task shifting the management of non-communicable diseases to nurses in Kibera, Kenya: does it work? *PLoS One* 2016, 11(1):e0145634.

38. Bukenya, D., Van Hout, MC ., Shayo, E., Kitabye, I., Musenze, B., Kasidi, J.R., Jaffar, S., Seeley, J. Accessing Integrated Health Care Services for HIV, Diabetes Mellitus and Hypertension in Selected Health Facilities in Kampala and Wakiso Districts, Uganda. *PLOS Global Health*. 2021; Early Online . DOI: https://doi.org/10.1371/journal.pgph.0000084

39. Van Hout, MC., Zalwango, F., Akugizibwe, M., Namulundu Chaka, M., Bigland, C., Birungi, J., Jaffar, S., Bachmann, M., Murdoch, J. (2022). Women living with HIV, diabetes and/or hypertension multimorbidity in Uganda: A qualitative exploration of experiences accessing a *'one stop'* integrated care service. *Journal of Integrated Care.* 2022, Early Online. https://www.emerald.com/insight/1476-9018.htm

40. Akugizibwe, M., Zalwango, F., Namulundu Chaka, M., Namakoola, I., Birungi, J., Okebe, J., Bachmann, M., Murdoch J., Jaffar, S., Van Hout, MC. "After all, we are all sick" : Multi-stakeholder understandings of stigma associated with integrated management of HIV, Diabetes and Hypertension at selected government clinics in Uganda. *BMC Health Services Research.* 2023, 23(20). https://doi.org/10.1186/s12913-022-08959-3

41. Shayo, E., Kivuyo, S., Seeley, J., Bukenya, D., Karoli, P., Mfinanga, S., Jaffar, S., Van Hout, MC. The acceptability of integrated healthcare services for HIV and non-communicable diseases: Experiences from patients and healthcare workers in Tanzania. *BMC Health Services Research* 2022, 22, 655 https://doi.org/10.1186/s12913-022-08065-4

42. Vorkoper S, Kupfer LE, Anand N, Patel P, Beecroft B, Tierney WM, Ferris R, El-Sadr WM: Building on the HIV chronic care platform to address noncommunicable diseases in sub-Saharan Africa: a research agenda. *AIDS (London, England)* 2018, 32(Suppl 1):S107.

43. Bonghaseh, DT., Bigland, C., Van Hout, MC. Understanding knowledge, attitudes, perceptions and

For peer review only - http://bmjopen.bmj.com/site/about/guidelines.xhtml

practices towards cardiovascular disease risk and prevention in patients living with HIV in Sub-Saharan Africa: A synthesis of qualitative literature. *Journal of Sustainable Development Africa* 2022; 24(2):11-29.

44. Adeyemi O, Lyons M, Njim T, Okebe J, Birungi J, Nana K, Claude Mbanya J, Mfinanga S, Ramaiya K, Jaffar S *et al*: Integration of non-communicable disease and HIV/AIDS management: a review of healthcare policies and plans in East Africa. *BMJ Global Health* 2021, 6(5).
45. Juma K, Reid M, Roy M, Vorkoper S, Temu TM, Levitt NS, Oladepo O, Zakus D, Yonga G. From HIV prevention to non-communicable disease health promotion efforts in sub-Saharan Africa: a narrative review. *Aids* 2018, 32:S63-S73.
46. Lupafya PC, Mwagomba BLM, Hosig K, Maseko LM, Chimbali H. Implementation of policies and strategies for control of noncommunicable diseases in Malawi: challenges and opportunities. *Health Education & Behavior* 2016, 43(1_suppl):64S-69S.
47. Shayo, E., Garrib, A., Birungi, J., Kivuyo, S. Mfinanga, S., Nyirenda, M., Namakola, I., Ramaiya, K., Bachmann, M., Cullen, W., Lazarus, J., Gill, G., Van Hout, MC., Shiri, T., Bukenya, D., Snell, H., Nanfuka, M., Cuevas, L., Shimwela, M., Mutungi, G., Musinguzi, J., Mghamba, J., Mangozi, S., Mugisha, K., Jaffar, S., Smith, P., Sewankamo N. Ethical issues in intervention studies on the management of treatment of diabetes and hypertension in sub Saharan Africa. *BMJ Global Health.* 2020, 5(7), pp e002193.
48. Murtagh, S., McCombe, G., Bachmann, M., Garrib, A., Jaffar, -S-., Lazarus, J., Mfinanga, S., Ramaiya, K., Sewankamo, N., Van Hout, MC., Cullen, W. (2021). Integrating diabetes, hypertension and HIV care in Africa: Delphi consensus study on international best practice *BMC Health Services Research* 2021; 21, 1235. Early Online https://doi.org/10.1186/s12913-021-07073-0
49. McCombe, G., Lim, J., Van Hout, MC., Lazarus, J., Bachmann, M., Garrib, A., Jaffar, S., Mfinanga, S., Ramaiya, K., Sewankamo, N., Cullen, W. Integrating care for diabetes and hypertension with HIV care in sub-Saharan Africa: A scoping review. *International Journal of Integrated Care.* 2021; 22(1): 6, 1–22. DOI: https://doi.org/10.5334/ijic.5839
50. Singh S, Kirk O, Jaffar S, Karakezi C, Ramaiya K, Kallestrup P, Kraef C. Patient perspectives on integrated healthcare for HIV, hypertension and type 2 diabetes: a scoping review. *BMJ Open* 2021,

For peer review only - http://bmjopen.bmj.com/site/about/guidelines.xhtml

11(11):e054629.

51. Haldane V, Legido-Quigley H, Chuah FLH, Sigfrid L, Murphy G, Ong SE, Cervero-Liceras F, Watt N, Balabanova D, Hogarth S. Integrating cardiovascular diseases, hypertension, and diabetes with HIV services: a systematic review. *AIDS Care* 2018, 30(1):103-115.

52. Moyo M, Musekiwa A. Protocol for updated systematic review and meta-analysis on the burden of non-communicable diseases among people living with HIV in sub-Saharan Africa. *BMJ Open* 2022;12(5):e055895.

53. Kintu A, Sando D, Okello S, Mutungi G, Guwatudde D, Menzies NA, Danaei G, Verguet S. Integrating care for non-communicable diseases into routine HIV services: key considerations for policy design in sub-Saharan Africa. *Journal of the International AIDS Society* 2020, 23:e25508.

54. Mahomed OH, Asmall S. Development and implementation of an integrated chronic disease model in South Africa: lessons in the management of change through improving the quality of clinical practice. *International Journal of Integrated Care* 2015, 15.

55. Pfaff C, Scott V, Hoffman R, Mwagomba B. You can treat my HIV–But can you treat my blood pressure? Availability of integrated HIV and noncommunicable disease care in northern Malawi. *African Journal of Primary Health Care and Family Medicine* 2017, 9(1):1-8.

56. Rawat A, Uebel K, Moore D, Yassi A. Integrated HIV-care into primary health care clinics and the influence on diabetes and hypertension care: an interrupted time series analysis in Free State, South Africa over 4 years. *Journal of Acquired Immune Deficiency Syndromes* 2018, 77(5):476-483.

57. Mwagomba BLM, Ameh S, Bongomin P, Juma PA, MacKenzie RK, Kyobutungi C, Lukhele N, Mwangi KJM, Amberbir A, Klipstein-Grobusch K. Opportunities and challenges for evidence-informed HIV-noncommunicable disease integrated care policies and programs: lessons from Malawi, South Africa, Swaziland and Kenya. *AIDS* 2018, 32:S21-S32.

58. Fisher, E.B., Boothroyd, R.I., Elstad, E.A. *et al.* Peer support of complex health behaviors in prevention and disease management with special reference to diabetes: systematic reviews. *Clinical Diabetes and Endocrinology* 2017; 3, 4.

59. Egbujie BA, Delobelle PA, Levitt N, Puoane T, Sanders D, van Wyk B. Role of community health workers in type 2 diabetes mellitus self-management: a scoping review. *PLoS One.*

For peer review only - http://bmjopen.bmj.com/site/about/guidelines.xhtml

2018;13(6):e0198424.

60. Decroo T, Telfer B, Dores CD*, et al* Effect of Community ART Groups on retention-in-care among patients on ART in Tete Province, Mozambique: a cohort study *BMJ Open* 2017;7:e016800.

61. Hagey JM, Li X, Barr-Walker J, Penner J, Kadima J, Oyaro P, Cohen CR. Differentiated HIV care in sub-Saharan Africa: a scoping review to inform antiretroviral therapy provision for stable HIV-infected individuals in Kenya. *AIDS Care*. 2018 Dec;30(12):1477-1487.

62. Jaffar S, Amuron B, Foster S, Birungi J, Levin J, Namara G, Nabiryo C, Ndembi N, Kyomuhangi R, Opio A, Bunnell R, Tappero JW, Mermin J, Coutinho A, Grosskurth H; Jinja trial team. Rates of virological failure in patients treated in a home-based versus a facility-based HIV-care model in Jinja, southeast Uganda: a cluster-randomised equivalence trial. *Lancet.* 2009; 374(9707):2080-2089.

63. Van Hout M-C, Bachmann M, Lazarus JV, Shayo EH, Bukenya D, Picchio CA, Nyirenda M, Mfinanga SG, Birungi J, Okebe J.Strengthening integration of chronic care in Africa: protocol for the qualitative process evaluation of integrated HIV, diabetes and hypertension care in a cluster randomised controlled trial in Tanzania and Uganda. *BMJ Open* 2020, 10(10):e039237.

64. Mfinanga SG, Nyirenda MJ, Mutungi G, Mghamba J, Maongezi S, Musinguzi J, Okebe J, Kivuyo S, Birungi J, Van Widenfelt E. Integrating HIV, diabetes and hypertension services in Africa: study protocol for a cluster randomised trial in Tanzania and Uganda. *BMJ Open* 2021, 11(10):e047979.

65. Georgeu D, Colvin CJ, Lewin S, *et al*. Implementing nurse-initiated and managed antiretroviral treatment (NIMART) in South Africa: a qualitative process evaluation of the stretch trial. *Implementation Science* 2012;7:66.

66. Craig P, Dieppe P, Macintyre S, *et al*. Developing and evaluating complex interventions: the new medical Research Council guidance. *BMJ* 2008;337:a1655.

67. Moore G, Audrey S, Barker M, *et al*. Process evaluation in complex public health intervention studies. London: MRC Population Health Science Research Network 2014.

68. Linnan L, Steckler A. Process evaluation for public health interventions and research. An overview. In: Steckler A, Linnan L,eds. *Process evaluation for public health interventions and research*. 1 edn. San Francisco: Josset-Bass, 2002: 1–23.

69. Murdoch J, Curran R, Bachmann M, *et al*. Strengthening the quality of paediatric primary care: protocol

For peer review only - http://bmjopen.bmj.com/site/about/guidelines.xhtml

for the process evaluation of a health systems intervention in South Africa. *BMJ Global Health* 2018;3:e000945.

70. Bronfenbrenner U. Toward an experimental ecology of human development. *Am Psychol* 1977;32:513–31.
71. Bronfenbrenner U. *The ecology of human development: experiments by nature and design.* Cambridge, MA: Harvard University Press, 1979.
72. Hawe P, Shiell A, Riley T. Theorising interventions as events in systems. *Am J Community Psychol.*2009;43(3-4):267-76.
73. Murdoch, J., Curran, R., Cornick, R. *et al.* Addressing the quality and scope of paediatric primary care in South Africa: evaluating contextual impacts of the introduction of the Practical Approach to Care Kit for children (PACK Child). *BMC Health Services Research* 2020, 20, 479.
74. Meghani A, Ssemugabo C, Pariyo G, Hyder AA, Rutebemberwa E, Gibson DG: Curbing the rise of noncommunicable diseases in Uganda: perspectives of policy actors. *Global Health: Science and Practice* 2021, 9(1):149-159.
75. Katende D, Mutungi G, Baisley K, Biraro S, Ikoona E, Peck R, Smeeth L, Hayes R, Munderi P, Grosskurth H. Readiness of Ugandan health services for the management of outpatients with chronic diseases. *Tropical Medicine & International Health* 2015, 20(10):1385-1395.
76. Grimshaw JM, Zwarenstein M, Tetroe JM, *et al*. Looking inside the black box: a theory-based process evaluation alongside a randomised controlled trial of printed educational materials (theOntario printed educational message, OPEM) to improve referral and prescribing practices in primary care in Ontario, Canada. *Implementation Science* 2007;2:38.
77. Karlsson G. *Psychological qualitative research from a phenomenological perspective*. Almqvist & Wiksell International,1993.
78. Mitchell CJ. Typicality and the case study. In: Ellen PF, editor. *Ethnographic research: a guide to general conduct.* New York: Academic 1984:238-41.

For peer review only - http://bmjopen.bmj.com/site/about/guidelines.xhtml

Table 2: Profile of Tanzania and Uganda regarding prevalence of three chronic conditions (hypertension, diabetes, and HIV)

Indicators	Uganda	Tanzania	References
Income levels	Low income level (GNI per capita income of $840)	Middle (GDP: $1348 for 2023)	World Bank (2022). Global income classification issued on July 1, 2022. Available at https://www.worldbank.org/en/news/factsheet/2022/07/07/world-bank-country-classifications-by-income-level-uganda# Word Bank projection
Population size	49, 332,360	65,497,748	United Nations (2022). Worldometer. Available at www.worldometers.info/world-population/uganda-population/ Tanzania Census, 2022
HIV prevalence	6.2%	4.9%	The Uganda Population-Based HIV Impact Assessment (UPHIA) (2016-2017) (2018). Available at 3430•PHIA-Uganda-SS_NEW.v14.pdf (columbia.edu) THIS, 2017
Diabetes prevalence	4.6%	9.1%,	International Diabetes Federation (2021). Diabetes Atlas. Available at https://data.worldbank.org/indicator/SH.STA.DIAB.ZS?locations=UG Tanzania Step Survey, 2013
Hypertension prevalence	33.4%	26%	Majumdar U, Nanyonga Clarke R, Moran AE, Doupe P, Gadikota-Klumpers DD, Gidio A, et al. (2022). Hypertension screening, prevalence, treatment, and control at a large private hospital in Kampala, Uganda: A retrospective analysis. *PLOS Glob Public Heal*;2:e0000386. Tanzania Step Survey, 2013
Doctors density/100 000 population	0.1((2015).	3 (2014)	World Health Organization (2022). Global Health Workforce Statistics, OECD, supplemented by country data. Available at https://data.worldbank.org/indicator/SH.MED.PHYS.ZS?locations=UG

For peer review only

Table 2 Objectives, contextual features and data collection methods of INTE-COMM process evaluation

**Objective/research question 1:** *What are patients, health care providers' and lay health workers' perceptions and experiences of integrated management of HIV, Diabetes, and Hypertension care in the community and at the health facility level?*			
**Contextual level/feature**	**Data Collection Methods**	**Sources of information**	**Tool to be used**
**Meso** Time and resources required; utilisation of policies and protocols; organisation and process of monthly meetings; training, organisation and work allocation of nurse and lay health worker; implementation processes and adaptations to embed intervention within communities<br><br>**Micro** Individual patient pathways to integrated care; relationship between intervention and management of HTN, DM and HIV within everyday life	Interview	Service users (patients stable on treatment)	Interview guides
	Interview	Health care providers (health facility managers, physicians/clinician, nurses, lay health workers, patient club facilitators)	Interview guides
	Focus groups	Community members/ leaders (8-12 participants)per group	Focus Group guides
	Interview	Representatives from Patient clubs/groups/peer educators.	Interview guides
	Interview	INTE-COMM investigators	Interview guides
**Objective/research question 2:** *What key contextual and process factors influence patients' and healthcare providers' uptake and acceptability of integrated management of HIV, Diabetes, and Hypertension care in the community and at the health facility level?*			
**Macro** Wider cultural discourses of HIV, hypertension and diabetes (e.g. HIV stigma); availability of medicines; socio-economic, trained staff, structures where services are delivered<br><br>**Meso** Geographic and demographic profile of communities and facilities; medicines supply; workforce arrangements of main social actors involved in intervention delivery (i.e. nurses and lay workers, patients, carers, community leaders); local practices and protocols; patient access and flow through community; referral pathways; role of traditional healers; and the different sites and scenes which impact on how INTE-COMM is adapted and delivered (e.g. local area management meetings)<br><br>**Micro**-individual level: influence of privacy and confidentiality in accessing care, trust placed to those delivering the intervention, mobility factor, economic factor	Interview	Policymakers and Reps of NGOs (ministerial policymakers and provincial/regional/district level clinical/health senior management (Directors/ Programme managers/ Commissioners, Assistant Commissioner of NCDs; Commissioner/Programme manager of the National AIDS control program)	Interview guides
	Interview	Health care providers (health facility managers, physicians/clinicians, nurses, lay health workers, patient club facilitators) peer groups/educators, traditional healers<br>Service care users (patients stable on treatment)	Interview guides<br><br>Interview guides
	Focus groups	Community members/leaders (8-12 participants per group)	Focus Group guide
**Objective/research question 3:** *To evaluate implementation and theoretical fidelity of the intervention*			
**Meso** Main (i.e. monthly meetings, interactions at dispensaries) activities and subsidiary activities (e.g. additional training/meetings, receptionist screening of patients) of intervention delivery; and interactional arrangements of each activity: structural arrangement at the point of service delivery (friendly environment, privacy and confidentiality issues)	Non-participant observations	Service delivery processes, the flow of clinical procedures, the nature of services given, listening in patients' and health workers' conversations; freedom in patients entering the service point interactions, discussing and asking questions when waiting for the services	Structured field-notes
**Objective/research question 4:** *What factors can potentially influence the scaling up of integrated management of HIV, Diabetes, and Hypertension at the community and health facility level?*			
**Macro** Cultural and structural characteristics, networks and communication, and external policies and	Interview	Service users	Interview guides

For peer review only - http://bmjopen.bmj.com/site/about/guidelines.xhtml

incentives<br><br>**Meso** Community and facility resource constraints; drug supply and patient medicine clubs; referral pathways	Interview	Healthcare providers (health facility managers, physicians/clinicians, nurses, lay health workers, patient club facilitators)	Interview guides
	Interview	Representatives from Patient clubs/group/peer educators	Interview guides
	Focus Groups	Community leaders and community members (8-12 participants per group)	Focus Group guides
	Interview	Policymakers and Reps of NGOs (ministerial policymakers and provincial/regional/district level clinical/health senior management (Directors/ Programme managers/ Commissioners, Assistant Commissioner of NCDs; Commissioner/Programme manager of the National AIDS control program)	Interview guides

[i] RESPOND-AFRICA (Research Partnership for the Control of Chronic Diseases in Africa) www.lstmed.ac.uk/RespondAfrica
[ii] MOCCA (Management of Chronic Conditions in Africa) www.lstmed.ac.uk/research/departments/international-public-health/respond-africa/mocca
[iii] INTE-AFRICA (Integrating and decentralising HIV, diabetes and hypertension services in Africa) www.inteafrica.org

For peer review only

For peer review only - http://bmjopen.bmj.com/site/about/guidelines.xhtml

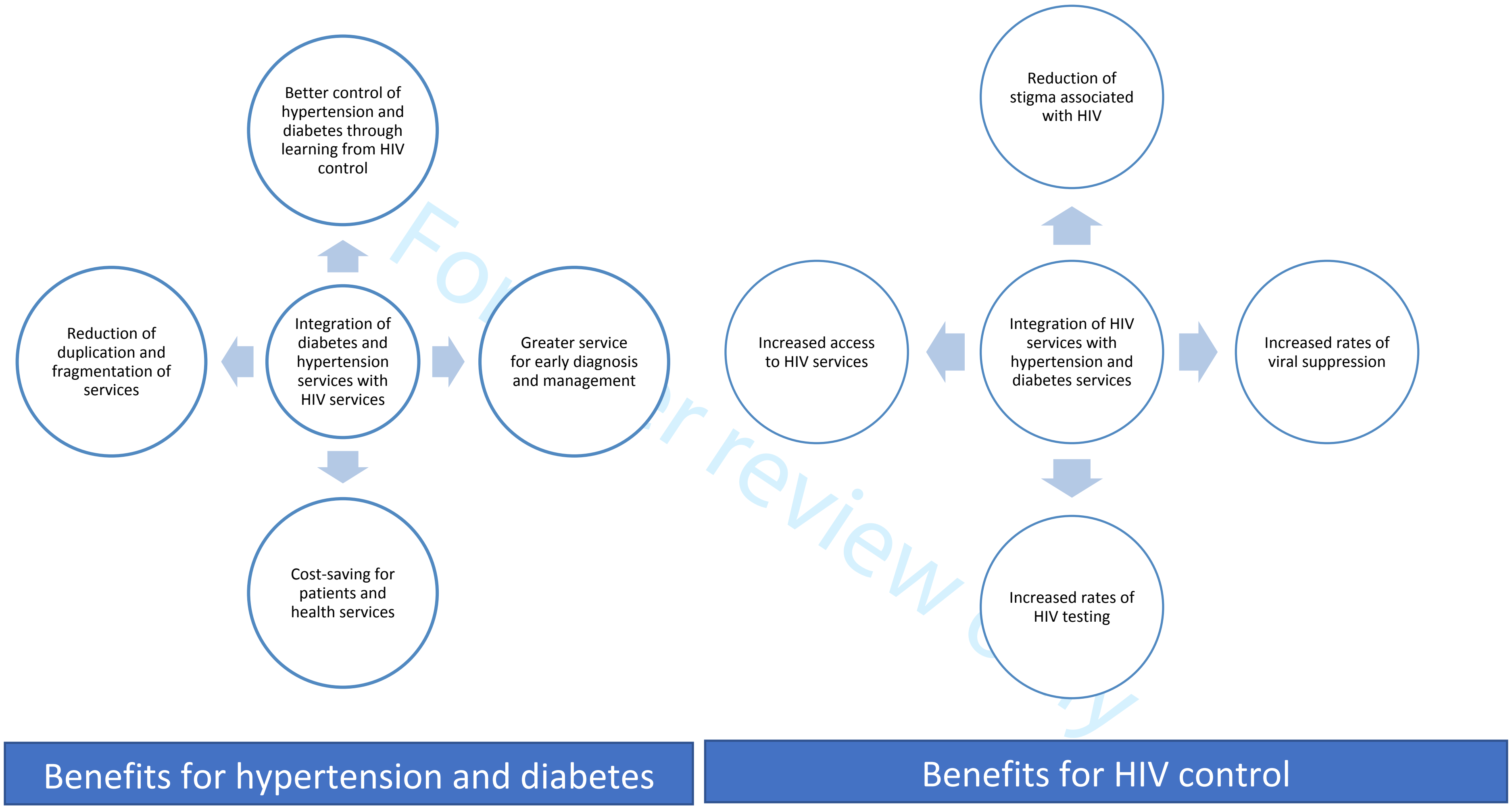

Figure 1: Benefits of integrated HIV, hypertension and diabetes care in general

For peer review only - http://bmjopen.bmj.com/site/about/guidelines.xhtml

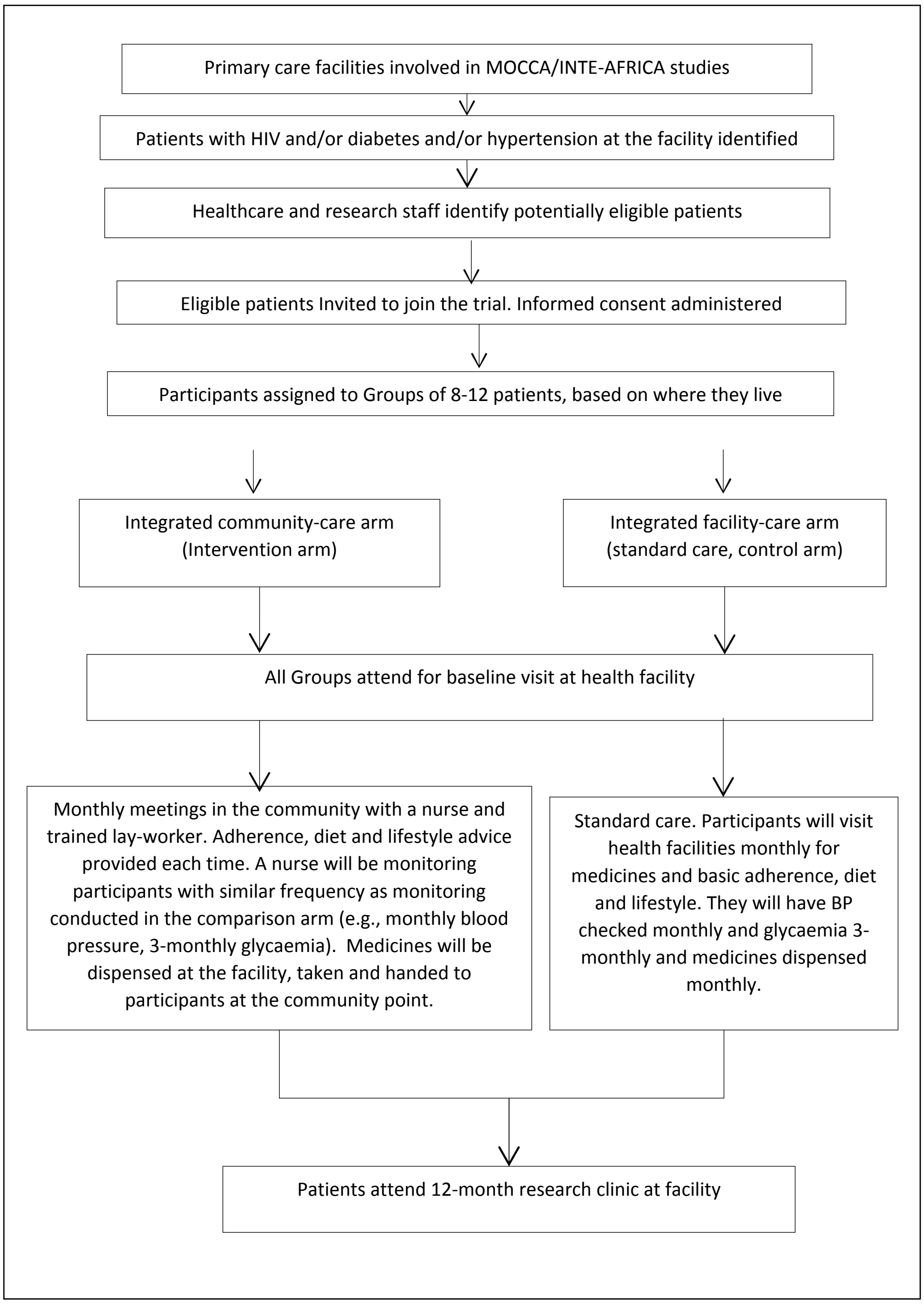

Figure 1. INTE-COMM study schema

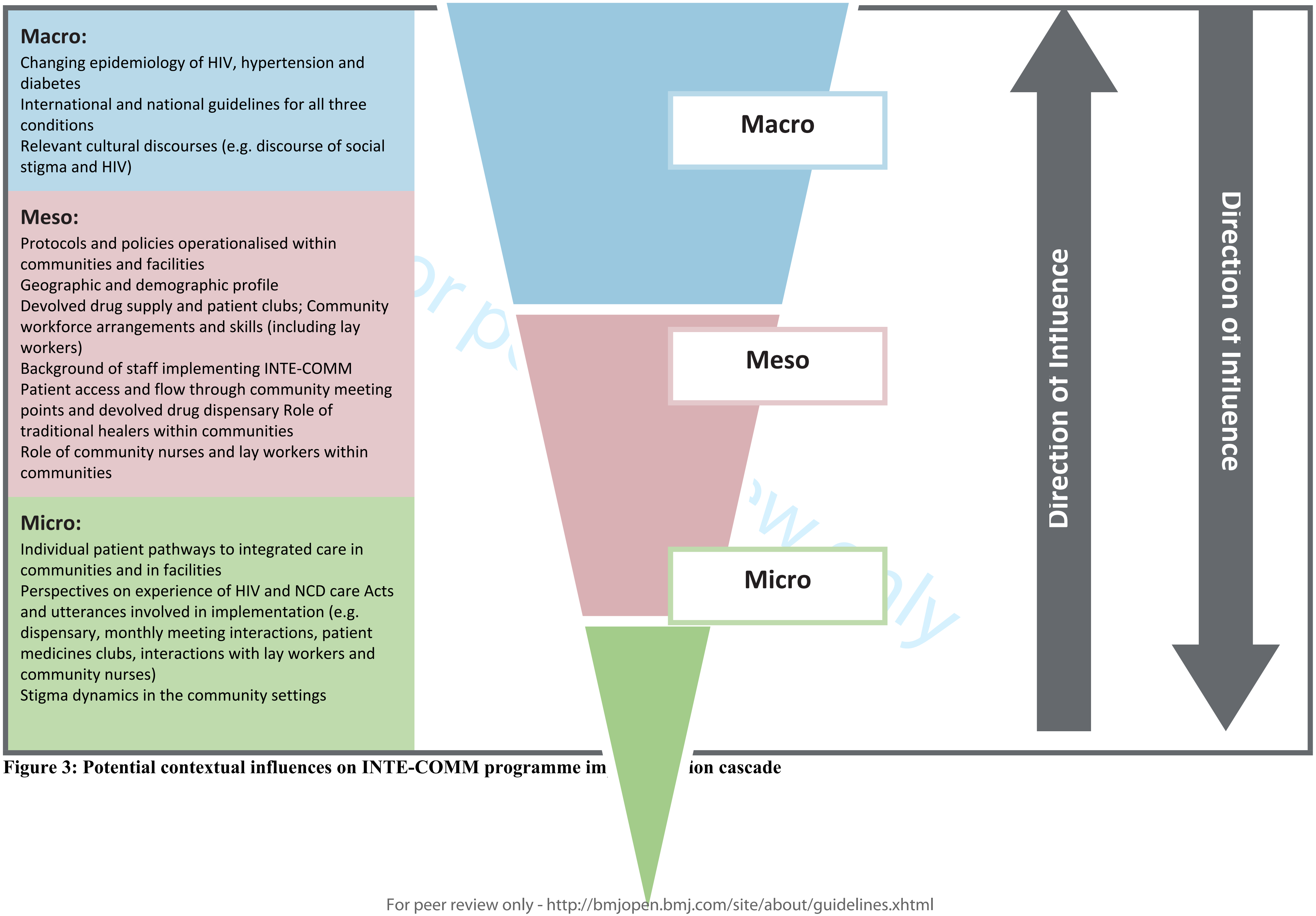

Figure 3: Potential contextual influences on INTE-COMM programme im[illegible]ion cascade

BMJ Open

Decentralizing chronic disease management in sub-Saharan Africa: a protocol for the qualitative process evaluation of community-based integrated management of HIV, diabetes, and hypertension in Tanzania and Uganda.

Journal:	*BMJ Open*
Manuscript ID	bmjopen-2023-078044.R1
Article Type:	Protocol
Date Submitted by the Author:	06-Feb-2024
Complete List of Authors:	Van Hout, Marie-Claire; Liverpool John Moores University, Public Health Institute<br>Akugizibwe , Mathias; MRC/UVRI and LSHTM Uganda Research Unit<br>Shayo, Elizabeth; National Institute for Medical Research, Policy Analysis and Advocacy<br>Namulundu , Moreen ; MRC/UVRI and LSHTM Uganda Research Unit<br>Kasujja, Francis; MRC/UVRI and LSHTM Uganda Research Unit<br>Namakoola, Ivan; MRC/UVRI and LSHTM Uganda Research Unit<br>Birungi, Josephine; MRC/UVRI and LSHTM Uganda Research Unit,<br>Okebe, Joseph; University College London<br>Murdoch, Jamie; King's College London, School of Life Course and Population Sciences,<br>Mfinanga, Sayoki; National Institute for Medical Research Tanzania, Muhimbili Medical Research Centre; NIMR,<br>Jaffar, Shabbar; University College London, Global Health
**Primary Subject Heading**:	Cardiovascular medicine
Secondary Subject Heading:	HIV/AIDS, Health services research, Infectious diseases
Keywords:	DIABETES & ENDOCRINOLOGY, Hypertension < CARDIOLOGY, HIV & AIDS < INFECTIOUS DISEASES

SCHOLARONE™
Manuscripts

Title Decentralizing chronic disease management in sub-Saharan Africa: a protocol for the qualitative process evaluation of community-based integrated management of HIV, diabetes, and hypertension in Tanzania and Uganda.

Abstract

Introduction: Sub Saharan Africa continues to experience a syndemic of human immunodeficiency virus (HIV) and non-communicable diseases (NCDs). Vertical (stand-alone) HIV programming has provided high-quality care in the region, with almost 80% of people living with HIV in regular care, and 90% virally suppressed. Whilst integrated health education and concurrent management of HIV, hypertension and diabetes is being scaled up in clinics, innovative, more efficient and cost-effective interventions which include decentralisation into the community are required to respond to increased burden of co-morbid HIV/NCD disease.

Methods and analysis: This protocol describes procedures for a process evaluation running concurrently with a pragmatic cluster-randomized trial (INTE-COMM) in Tanzania and Uganda which will compare community-based integrated care (HIV, diabetes, hypertension) with standard facility-based integrated care. The INTE-COMM intervention will manage multiple conditions (HIV, hypertension, diabetes) in the community via health monitoring and adherence/lifestyle advice (medicine, diet, exercise) provided by community nurses and trained lay-workers, and the devolvement of NCD drug dispensing to community level. Based on Bronfenbrenner's ecological systems theory, the process evaluation will use qualitative methods to investigate socio-structural factors shaping care delivery and outcomes in up to 10 standard care facilities and/or intervention community sites with linked healthcare facilities. Multi-stakeholder interviews (patients, community health workers/volunteers, healthcare providers, policymakers, clinical researchers, international and non-governmental organisations), focus group discussions (community leaders, members) and non-participant observations (community meetings, drug dispensing) will explore implementation from diverse perspectives at three timepoints in the trial implementation. Iterative sampling and analysis moving between data collection points and data analysis to test emerging theories will continue under saturation is reached. This process of analytic reflexivity and triangulation across methods and sources

For peer review only - http://bmjopen.bmj.com/site/about/guidelines.xhtml

will provide findings to explain the main trial findings and offer clear directions for future efforts to sustain and scale up community-integrated care for HIV, diabetes and hypertension.

Ethics and dissemination: The protocol has been approved by the University College of London (UK), London School of Hygiene and Tropical Medicine Ethics Committee (UK), Uganda National Council for Science and Technology and the Uganda Virus Research Institute Research and Ethics Committee (Uganda), and the Medical Research Coordinating Committee of the National Institute for Medical Research (Tanzania) . University College of London is the trial sponsor. Dissemination of findings will be done through journal publications and stakeholder meetings (study participants, healthcare providers, policy makers and other stakeholders), local and international conferences, policy briefs, peer reviewed journal articles and publications.

Trial registration number ISRCTN15319595. Pre-results.

For peer review only

Strengths and Limitations: Summary Box

The INTE-COMM trial responds to the continued need to tackle the increased burden of NCDs in sub-Saharan Africa, and decentralise healthcare systems into the community to manage co/multi-morbidities (diabetes/hypertension) with HIV.

A pragmatic cluster-randomized trial will compare community-integrated care for HIV, diabetes and hypertension with standard facility-based integrated care for the first time in Tanzania and Uganda.

The INTE-COMM trial is based on a partnership between African and UK researchers, working closely with policy-makers, service user representatives, healthcare providers and community members to provide integrated care for people living HIV, diabetes or hypertension in the community.

The process evaluation of INTE-COMM employs qualitative interviews and observational methods at 4-10 sites to explore and document delivery of integration care in the community setting, with a view to understanding the role of individual, community, familial, social, structural factors shaping implementation and outcomes.

Foreseen limitations of the process evaluation may include community site characteristics, lack of service user and community buy-in due to perceptions that community care is inferior to facility -based integrated care, stigma, lack of trust in community drug dispensing issues, service user drop-out, social desirability bias and various other contextual mitigating factors.

Key Words

HIV, non-communicable disease (NCD), sub-Saharan Africa, diabetes, hypertension, decentralisation, community integrated care, Tanzania, Uganda

.

For peer review only

INTRODUCTION

The global burden of non-communicable diseases (NCDs) is recognised in the Sustainable Development Agenda 2023 and the sustainable development goals (SDG) as a substantial challenge for sustainable development[1]. Most recent global NCD data from the 2019 Global Burden of Disease Study has reported that NCDs kill 41 million people annually (equivalent to 74% of all deaths globally). This study has also documented a concerning global increase in NCD deaths over time, with the percentage of deaths due to NCDs increasing from 60% in 1999 to 74% in 2019 (approximately 15% increase over two decades)[2]. The majority of NCD deaths are due to cardiovascular diseases (estimated 17.9 million annually), followed by cancers (9.3 million), chronic respiratory diseases (4.1 million), and diabetes (2 million which includes kidney disease deaths due to diabetes)[1]. 17 million people are reported to die from an NCD before the age of 70 years[1-2].

NCDs present enormous challenges to progress towards attaining the targets set in the Sustainable Development Agenda 2030. The SDG target (3.4) aims to reduce by one third premature mortality from NCDs through prevention and treatment by 2030[1]. Prevention, screening, treatment and care of NCDs (including palliative care) are key components of the NCD response[1]. Various modifiable behavioural risk factors increase the risk of NCD related mortality (e.g. tobacco and alcohol use, excess salt intake and unhealthy diets, physical inactivity). Metabolic risk factors such as obesity, hyperglycaemia, hypolipidemia, hypertension) increase risk of NCD disease[1-2]. Other environmental factors such as air pollution cause NCDs such as lung cancer, chronic obstructive pulmonary disease, ischaemic heart disease and stroke[1-2].

Low and middle income countries (LMICs) are however disproportionately impacted by NCD morbidity and mortality[3-6]. Poverty reduction initiatives are hindered by rising HIV/NCD multi-morbidities due to increased household costs related to healthcare, and higher mortality and morbidity of the vulnerable and socially disadvantaged[1]. Of all NCD deaths, 77% are in LMICs, where 86% of NCD deaths before the age of 70 years occur[1-2]. The Global Alliance of Chronic Diseases also observes that of the more than 15 million people aged 30-69 years who die annually due to NCDs, with 85% of these premature deaths occur in LMICs. Hypertension and diabetes cause the most NCD related mortality and morbidity in LMICs[7-8]. Women living in LMICs are also disproportionately affected

For peer review only - http://bmjopen.bmj.com/site/about/guidelines.xhtml

by the triple burden of reproductive and maternal health conditions, NCDs, and human immunodeficiency virus (HIV)[4-6].

Sub-Saharan Africa

The sub-Saharan African (SSA) region continues to experience a syndemic of HIV and NCDs[1,9-14]. The region accounts for 55% of the total 38 million people living with HIV (PLHIV) globally[15]. In addition to the rise in patients in regular HIV care (almost 80%) and virally suppressed (90%),[16] NCDs among PLHIV in SSA are also increasing. This is due to various HIV related reasons (HIV infection itself, anti-retroviral treatment (ART)); and aging related chronic co-morbidities (diabetes, hypertension, cancers, metabolic disorders)[17-20]. Key relevant socio-economic factors fuelling the NCDs rise in the region are increased urbanisation, poor lifestyle and poverty, NCD related healthcare costs (e.g. drugs) and with vulnerable and poor populations substantially impacted by chronic illness[9,11-12,21].

Long term management of diabetes and hypertension among PLHIV remains a major health system challenge in SSA[22-23] where rising HIV/NCD multi-morbidity and earlier age of onset of disease of diabetes and hypertension are observed[11-12,14,24-27]. In response to this, efforts to coordinate NCD programmes with scaled up NCD medicine supply chains alongside high quality vertical (stand-alone) HIV programmes have intensified[28]. There is a growing evidence base and shift toward the implementation of integrated NCD/HIV care provision in various SSA countries (e.g. in Malawi, South Africa, Botswana, Uganda, Kenya, Tanzania)[22,29-41]. Integrated health education and concurrent management of HIV, hypertension and diabetes is being scaled up in vertical and One Stop clinics in the region, and can reduce duplication and fragmentation of services; support medicine supply chains, streamline detection and care of co/multi-morbidities; support patient uptake, retention and adherence to treatment; increase viral suppression rates and achieve better control of hyperglycaemia/hypertension; encourage health awareness raising, and reduce HIV related stigma[21,42-57]. See **Figure 1.**

Insert **Figure 1: Benefits of integrated HIV, hypertension and diabetes care in general** about here

Innovative, more efficient and cost-effective interventions explicitly focused on tackling the HIV, hypertension and diabetes co/multimorbidity's in SSA community (particularly rural) settings are

For peer review only - http://bmjopen.bmj.com/site/about/guidelines.xhtml

required to respond to increased burden of NCDs and co/multi-morbidities. Whilst community or home based care of HIV using outreach, lay health workers and peer support models is expanding in various SSA countries (Uganda, Mozambique, Kenya), community models diabetes and hypertension care are less developed[58-62]. Our Research Partnership for the Control of Chronic Diseases in Africa (RESPOND-AFRICA) partnership will generate evidence specific to community-based integrated care of HIV, diabetes and hypertension in two SSA countries which can be used to inform and support decentralisation of integrated care for these conditions. We present here the process evaluation of a pragmatic cluster-randomized trial (INTE-COMM) in Tanzania and Uganda which will compare community-integrated care (HIV, diabetes, hypertension) with standard facility-based integrated care (protocol number ISRCTN15319595).

The INTE-COMM Project

RESPOND-AFRICA has operated in Tanzania and Uganda for many years. We have piloted integrated HIV/NCD care in a feasibility single arm intervention study in Uganda, and in Tanzania in the Management of Chronic Conditions in Africa (MOCCA) project (2018-2020)[21,29,38,41]. MOCCA was followed by scaled up integrated care for HIV, diabetes and hypertension in the Integrating and decentralising HIV, diabetes and hypertension services in Africa (INTE-AFRICA trial) (2019-2023)[39-40,63-64]. INTE-COMM is a 4-year research project funded by the National Institute for Health and Care Research (NIHR) (01/10/2020-30/09/2024). We will closely adhere to the Medical Research Council (MRC) framework for developing and evaluating complex interventions and frameworks for intervention adaptation and multimorbidity interventions. In a formative phase of 18 months, we have been guided by community-based models of HIV care that include provision of medicines, peer-support and self-management, and a series of collaborative stakeholder consultations with policy makers and patient (service user) groups to design a new community-based integrated model of care for the management of patients with HIV, diabetes and hypertension. This has informed the design of INTE-COMM which will differ from existing community care approaches by managing multiple conditions (HIV, hypertension, diabetes) in the community via health monitoring and adherence/lifestyle advice (medicines, diets, exercise) provided by community nurses and trained lay workers, and devolve NCD drug dispensing to the community level. Whilst INTE-COMM is situated

For peer review only - http://bmjopen.bmj.com/site/about/guidelines.xhtml

in Tanzania and Uganda, we intend to generate evidence potentially transferable to other community settings in SSA and wider continent.

Process Evaluation of the INTE-COMM trial

Process evaluations typically evaluate how and whether interventions are delivered as intended and whether such implementation is congruent with the theory underpinning the intervention[65-69]. The MRC guidance for evaluation of complex health interventions underscores the importance of process evaluation within trials[66]. Building on experiences and lessons learnt in our process evaluation of the INTE-AFRICA trial[63], the process evaluation of INTE-COMM will focus explicitly on assessing the various system dimensions pertinent to the community care context; e.g. how the meanings of HIV, diabetes and hypertension in communities influence receipt of the INTE-COMM intervention; how implementation processes function to facilitate or inhibit access to care; and the mechanisms driving service user/ provider and community outcomes.

Using qualitative interviews and non-participant observations we will document and explore the experiences, attitudes and practices of a wide variety of stakeholders during implementation in order to develop a process-led understanding of how socio-structural factors shape initiation, delivery and implementation of community-based HIV/NCD service integration over time. We will clarify causal mechanisms and contextual factors associated with variation in outcomes between community and facility-based integrated care; and assess to what extent resources and activities are supporting the INTE-COMM trial in delivering intended outputs and related clinical and health economics outcomes.

A central focus lies in identifying challenges and contextually relevant strategies or solutions in the community setting which can support and guide successful implementation and sustainability of community-based integrated prevention, detection and management of HIV with hypertension and diabetes. We are cognisant that this community approach is not devoid of controversy as service users will be moved away from traditional facility-based integrated care where they were managed by certified clinicians to a community venue where services are delivered by a trained lay worker supported by a nurse. Patients can perceive this as inferior and a far cry from those managed in standalone specialist facilities. We also appreciate that HIV is a stigmatising condition and putting patients together with others without the condition in a community setting may be disconcerting. These potential

For peer review only - http://bmjopen.bmj.com/site/about/guidelines.xhtml

limitations to effective delivery of the intervention will be carefully monitored within this process evaluation, and insights used to both explain trial findings and inform future wide-scale implementation.

Theoretical framework: Ecological systems theory

The process evaluation will adapt Bronfenbrenner's ecological systems theory[70-71] to conceptualise the INTE-COMM intervention as an event which disrupts complex social systems operating across multiple contextual levels[67,72]. The theory provides an organising structure to facilitate in-depth understanding of how implementation of the intervention is shaped by the wider context, and importantly for this study, it's interaction with the local communities into which INTE-COMM is introduced[63,67,70-73]. Drawing on our previous primary care research in LMIC settings[73], three elements of context, (see **Figure 2**), will be investigated to capture variation in adoption, delivery and maintenance which are likely to be important factors in outcome differences.

Insert **Figure 2: Potential contextual influences on INTE-COMM programme implementation cascade** about here

METHODS AND ANALYSIS

Study setting

Tanzania and Uganda were chosen as study sites given their pioneering work in this area[29,38-40,62-63,74-75]. Their public health services are strongly committed to providing services for NCDs, and are struggling to scale-up provision for diabetes and hypertension in the face of competing health demands, including from HIV and diabetes and hypertension across all socioeconomic strata[74]. See **Table 1**.

Insert **Table 1: Profile of Tanzania and Uganda regarding prevalence of three chronic conditions (hypertension, diabetes, and HIV)** about here

Study population and recruitment

Patients with HIV, diabetes, or hypertension who are considered stable on treatment at the health facilities will be invited to join the INTE-COMM trial. The trial will evaluate whether community-based integrated care will be effective in improving patient outcomes among PLHIV, diabetes, and hypertension as compared to standard facility-based integrated care (and including outcomes regarding uptake, retention, acceptability, costs, linkage and potential cost-effectiveness of integrated community care). Patients living with HIV, diabetes, or hypertension, stable on treatment at facilities, will be

For peer review only - http://bmjopen.bmj.com/site/about/guidelines.xhtml

organized into groups of approximately eight-14 persons and randomized to community-based or facility-based integrated care. The study has two co-primary endpoints; a composite endpoint of glycemia and blood pressure control among individuals with diabetes and/or hypertension and plasma viral load suppression among PLHIV. In the integrated community arm, participants will receive their drugs, adherence support, health education and monitoring at a community venue. Facility based integrated care will comprise of combined health education and clinical management of HIV, diabetes and hypertension. See **Figure 3.**

Insert **Figure 3. INTE-COMM study schema** about here

All trial participants will be followed up for 12 months. A sample size of 116 groups consisting of 12 persons; of which eight will have diabetes or hypertension and four with HIV (total of 1,392 participants) will provide over 80% power to detect an absolute difference in blood pressure and blood glucose control of 10% at the 5% two-sided significance level. For HIV viral suppression, the trial will have over 80% power to show non-inferiority with a margin of delta= 8.5%, 7.5%, and 5.5% assuming viral suppression is 85%, 90%, and 95% respectively. To allow for loss to follow up of over 10% in the groups, we will recruit 124 groups, comprising 14 persons, giving a target sample of 1,736 participants. Each country will enrol a total of 62 groups and 868 participants into the trial (protocol reported elsewhere).

A purposive sampling approach will recruit process evaluation participants. Recruitment will continue until data saturation is reached (see analysis). Service users recruited from those who have already consented to participate in the main trial from both arms, of both genders and on stable treatment for either hypertension, diabetes, HIV, or multi-morbidity will be recruited for face-to-face interview with the help of gatekeepers (facility and community nurses) at three sites (urban, rural and peri-urban) (estimated n=28 in each country). Replacement of participants (e.g. service users) will occur if significant loss to follow-up or refusal of repeated interviews occurs. Healthcare service providers at both arms (health facility managers, physicians/clinicians, nurses, lay workers, patient club facilitators/peer educators and traditional healers) will also be invited for face-to-face interview (estimated n= 5-10 in each country). Key ministerial policymakers and provincial/regional/district level clinical/health senior management (Director/programme manager/Commissioners, Assistant

For peer review only - http://bmjopen.bmj.com/site/about/guidelines.xhtml

Commissioner of NCDs; Programme manager/ Commissioner of the National AIDS Control programme and others), non-governmental and international organisations representatives will be identified based on the INTE-AFRICA listing and will be invited for face to face or telephonic interview where it is difficult to meet physically (estimated n=5-10 in each country). Community leaders and members will be identified by gatekeepers in the facility catchment areas and invited to partake in three focus group discussions with six-10 participants (female, males, community leaders of both genders) with the help of Village Health Team members. Clinical researchers in the INTE-COMM trial will be interviewed in both countries and asked to provide reflections on the implementation process.

Design of Process Evaluation

This protocol describes procedures for a process evaluation using qualitative methods within a pragmatic cluster-randomized trial which will compare community-integrated care with facility-based integrated care in Tanzania and Uganda. Qualitative interviews and non-participant observations will be conducted concurrently with the trial, and in tandem with the collection of clinical outcomes and health economic data. Qualitative methods will include multi-stakeholder interviews (patients, community health workers/volunteers, healthcare providers, policymakers, clinical researchers, international and non-governmental organisation), focus group discussions (community leaders, members), supported by observations of community group meetings in intervention sites. Data collection using these methods will document and explore various dimensions of implementation from diverse perspectives at three timepoints in the trial implementation (at the baseline, at six months, and at 12 months). See **Table 2** for further detail according to detailed research objectives per participant group.

Insert **Table 2 Objectives, contextual features and data collection methods of INTE-COMM process evaluation** about here

By using Bronfenbrenner's ecological systems model, this design permits an assessment of theoretical fidelity of INTE-COMM's implementation, understood for how the intervention is flexibly adapted to function within localised community contexts, rather than a strict assessment of fidelity[67-68]. To do so requires a detailed description of the processes, relationships, and contexts involved in the delivery of community-based integrated care, and the identification of factors attributing to the

For peer review only - http://bmjopen.bmj.com/site/about/guidelines.xhtml

failure or success of the programme. It addresses the black box problem in interpreting trial results by improving understanding of the mechanisms that connect particular intervention components to particular outcomes[76]. This synergistic approach to evaluate, understand and respond will enhance acceptability of community level integrated approaches in both countries to support refinement of care for HIV, diabetes and hypertension, inform health surveillance, support effective drug dispensing, staff capacity building and future policy reforms to involve and educate local communities, sustain and scale up integrated HIV/diabetes/hypertension care in Tanzanian and Ugandan community settings.

Data collection

Data collection tools (interview and focus group guides) will explore multi-stakeholder perceptions and experiences of the decentralized integrated management of HIV, diabetes, and hypertension care in community and facility settings. Interview and focus group guides will be designed based on a scoping review of literature and process evaluation team consultation with the INTE-COMM trial implementers. Questions will be carefully developed in order to develop a phenomenological understanding[77] of the experiences, processes and contextual factors that influence the implementation of the intervention within community eco-and cultural systems (see Table 2 research objectives and supplemental files). Questions and probes will unpack and identify various factors that can potentially influence the sustaining and scaling up of integrated management of HIV, diabetes, and hypertension in the community and at facility levels will be identified and explored. We include an explicit objective to monitor and identify potential sources of contamination and dilution of the intervention, for example relating to HIV and whether people in intervention communities are going elsewhere.

Where possible, we will gender-match the interviewer with participant, particularly in the case of service users. In Tanzania, all interviews and focus groups will be conducted in Swahili language, in Uganda interviews with service users and focus groups with community members/leaders of medicine clubs will be conducted in local languages and the remainder will be conducted in English. Data collection tools will therefore be translated accordingly. Audio recordings will be taken with written and verbal consent and transcribed verbatim by the team. Back translation of transcripts from the local language into English will occur for consistency. Non-participant observations of community group

For peer review only - http://bmjopen.bmj.com/site/about/guidelines.xhtml

For peer review only

meetings will be documented using written field notes to provide a description of the processes followed and the content involved in delivering integrated care.

Data analysis and synthesis

Iterative sampling and analysis moving between data collection points and data analysis to test emerging theories will continue under saturation is reached. This process of analytic reflexivity and triangulation across methods and sources will provide findings to explain the main trial findings and offer clear directions for future efforts to sustain and scale up community-integrated care for HIV, diabetes and hypertension.

Using this reflexive iterative approach which we have developed in our previous health systems research[63,73] we will analyse both theoretical fidelity and the interaction between the INTE-COMM intervention, local communities and the wider health system context. This will be achieved by: 1) setting out relevant macro (e.g. cultural discourses of NCDs); meso (e.g. protocols used in monthly meetings, structural arrangements at service points to observe privacy and confidentiality, lay health worker/nurse arrangements); and micro (e.g. talk and behaviour at dispensaries) contextual features relevant to implementation; 2) targeting where likely tensions in implementation are likely to occur at each contextual level; then 3) analysing tensions within targeted activities involved in intervention delivery; and 4) considering the consequences of these tensions for how the intervention was implemented and the implications of these for scaled up implementation. Tensions in implementation are likely to be manifested in training sessions, interactions between lay health workers, peer educators, nurses and members of the community, or through organisational processes for ensuring the intervention is delivered.

To manage the quantity and range of data collected as part of the process evaluation, iterative analysis will involve working laterally across data types, collection points and situating evidence within the macro, meso, micro contextual framework (see Table 2). We will seek to provide a broad description of intervention delivery but, instead of allocating equal time to the analysis of each case, we will focus on identifying '*telling cases',* triangulating and looking for connections between data[78]. Care will be taken to identify and follow up deviant cases which do not fit into emerging theories. Emerging theories and the relationship of the data to the conceptual literature underpinning the intervention will be

For peer review only - http://bmjopen.bmj.com/site/about/guidelines.xhtml

discussed and refined at team meetings throughout the research. Inductive (iterative) analysis will further unpack how service users, providers, other stakeholders and their communities understand and apply diverse lay health literacies, autonomies and decision-making processes including health care seeking regarding the three conditions of interest (HIV, diabetes and hypertension) and settings of clinical care[63,67,76]. An electronic data management package (NVivo.14) will support the coding processes (including pilot coding of a small number of transcripts at the beginning) undertaken by the research team. This process is supported by regular briefing meetings and team discussion around theme allocation, consensus and explanation of potential outliers.

Credibility and transferability

The process evaluation protocol adheres to recommendations intended to facilitate the standardisation of process evaluation design and reporting[67]. The credibility and reliability of our data will be anchored in the ability to triangulate socio-behavioural and observational data during iterative processes of collection and analysis in order to better understand how different types of evidence enhance overall interpretation of community-based integrated care. Triangulation will occur across theories, methods (qualitative, observations) between countries (Tanzania and Uganda) and sources of data (narratives, checklists, notes).

The process evaluation also operates concurrently with clinical outcomes data and health economics analysis. Taken collectively process evaluation data with clinical and health economics data can inform scalability and transferability to other community settings.

We recognise the potential for selection and information bias as limitations of the trial itself, and mitigate by using a standardised approach to collecting data in an iterative process with continual assessment of information bias, and ensuring that research personnel are unaware of participant disease status. Social desirability is addressed in the process evaluation by only providing brief information at the outset of the evaluation in order to avoid priming, using an interview schedule approved by a panel of INTE-COMM experts in terms of sensitivity, conducting qualitative research using skilled local interviewers with limited power relationship between interviewer and participant, using safe and secure settings where the participant feels comfortable or using technology/telephone, briefing them that there

For peer review only - http://bmjopen.bmj.com/site/about/guidelines.xhtml

is no right and wrong answer, and finally by encouraging them to use anecdotes and experiential evidence to support their views.

Patient and public involvement

As with all RESPOND-AFRICA projects, proactive community engagement is crucial for the success of INTE-COMM. The INTE-COMM trial is cognisant of the importance of public and patient involvement (PPI) throughout its programme of research, and values PPI in enhancing research quality and relevance by providing different perspectives and a sense of ownership. The process evaluation will allow multi-stakeholder voices to be heard, and utilised. Key community level stakeholders such as service users and their families, community members and community care professionals will be fully involved in guiding the research, acting as research participants, and in implementation of change in community level integrated health service delivery and integrated care planning. All aspects of the process evaluation are underpinned by participatory action health research and its success and usefulness will be grounded in PPI, participation and engagement in the form of patient/professional identification of research priorities, collaborations and partnerships, expert steering, community participation around health needs and optimal integrated services, awareness raising activities, development of print materials, toolkits and training for community nurses, lay members and healthcare professionals.

ETHICS, DATA MANAGEMENT AND DISSEMINATION

The protocol has been approved by the University College of London (United Kingdom), London School of Hygiene and Tropical Medicine Ethics Committee (United Kingdom), Uganda National Council for Science and Technology and the Uganda Virus Research Institute Research and Ethics Committee (Uganda) and from the Medical Research Coordinating Committee of the National Institute for Medical Research (Tanzania). University College of London, United Kingdom is the trial sponsor. The key ethical principles of voluntary and informed participation, respect of persons, do no harm, autonomy, privacy, anonymity, confidentiality, and safety of participants will be used in all researcher and participant interactions. Written informed consent for participation in interviews and focus groups will be obtained from all participants. All participants will be provided with written information about

For peer review only - http://bmjopen.bmj.com/site/about/guidelines.xhtml

the research, this will be explained verbally, and informed that their participation is voluntary and that they may withdraw from participation at any time without any penalty.

In terms of data management, safety of all data will be ensured by: (1) applying encrypted password-protected protected code to all transcripts; (2) storage of all data in an encrypted database for up to 10 years and only be accessible by the INTE-COMM research teams in Tanzania, Uganda and the United Kingdom; (3) storing all personal identifying information of participants separately from all transcripts and allocating each participant with a unique participant identification number to delink the information from the personal identifiers; and (4) destroying all audio recordings on completion of transcription. The transcript files will be uploaded onto the encrypted database where they will be stored for up to 10 years and thereafter destroyed by the trial team. Across INTE-COMM countries, only anonymised participants' data will be shared during routine updates and briefing sessions. No personal identifying information (for example consent forms and audio recordings) will be shared across countries or via any communication means.

Timely communication and publication of findings across a broad range of audiences is essential to drive policy considerations around community-based integrated HIV/NCD care in Africa. Through targeted dissemination, communication, outreach and provision of innovative tools and models, the project's long-term conceptual, instrumental, capacity building and network influences will centre on achieving an enhanced understanding of the intricacies involved in integrating HIV and NCD service and patient care at the community level. Dissemination and communication will fully and regularly utilise all established and innovative channels (for example journal publications, conferences, press releases, webinars) target all stakeholders, including a variety of audiences and in local languages and English.

Authors Contributions

All authors contributed to the conceptualisation of the research and contributed to writing the manuscript.

MCVH, MA, EHS, MN, IN, JM, AK, GSM, SJ designed the process evaluation protocol.

SJ, JO, FK, JB led the development of the INTE-COMM trial.

For peer review only

MCVH drafted the manuscript and all co-authors edited and commented on subsequent drafts. All authors approved the final draft for submission. All authors agree to be accountable for all aspects of the work in ensuring that questions related to the accuracy or integrity of any part of the work are appropriately investigated and resolved.

Funding Acknowledgement: 'This work was supported by NIHR; GHPSR Project: NIHR 131273

Competing interests: None

Patient consent: Not required.

Ethics approval: The protocol has been approved by the University College of London (UK), London School of Hygiene and Tropical Medicine Ethics Committee (UK), Uganda National Council for Science and Technology and the Uganda Virus Research Institute Research and Ethics Committee (Uganda), and the Medical Research Coordinating Committee of the National Institute for Medical Research (Tanzania).

Provenance and peer review: Not commissioned; internally peer reviewed.

Data sharing statement: No additional data are available.

Open access: This is an open access article distributed in accordance with the Creative Commons Attribution 4.0 Unported (CC BY 4.0) license, which permits others to copy, redistribute, remix, transform and build upon this work for any purpose, provided the original work is properly cited, a link to the licence is given, and indication of whether changes were made. See: https:// creative commons. org/ licenses/ by/ 4. 0/.

REFERENCES

1. World Health Organization (WHO). *Noncommunicable diseases.* World Health Organization, 2024. Available at www.who.int/news-room/fact-sheets/detail/noncommunicable-diseases (Accessed 25 January 2024)
2. Global Burden of Disease Collaborative Network. *Global Burden of Disease Study 2019* Available at https://vizhub.healthdata.org/gbd-results/
3. Das, M. WHO urges immediate action to tackle non-communicable diseases. *Lancet Oncology,* 2022. Early Online. DOI:https://doi.org/10.1016/S1470-2045(22)00606-4

For peer review only - http://bmjopen.bmj.com/site/about/guidelines.xhtml

4. Alwan A. *Global status report on noncommunicable diseases 2010.* World Health Organization, 2011.
5. Hoffman RM, Newhouse C, Chu B, Stringer JS, Currier JS. Non-communicable Diseases in Pregnant and Postpartum Women Living with HIV: Implications for Health Throughout the Life Course. *Current HIV/AIDS Reports* 2021, 18(1):73-86.
6. NCD Alliance. *A call to action: women and non-communicable diseases.* 2022. Available at Women's Health | NCD Alliance (Accessed 16 February 2023)
7. Adler AJ, Prabhakaran D, Bovet P, Kazi DS, Mancia G, Mungal-Singh V, Poulter N. Reducing cardiovascular mortality through prevention and management of raised blood pressure. *Global Heart* 2015, 10(2):111-122.
8. Bennett JE, Kontis V, Mathers CD, Guillot M, Rehm J, Chalkidou K, Kengne AP, Carrillo-Larco RM, Bawah AA, Dain K.NCD Countdown 2030: pathways to achieving Sustainable Development Goal target 3.4. *The Lancet* 2020, 396(10255):918-934.
9. Addo J, Smeeth L, Leon DA: Hypertension in sub-saharan Africa: a systematic review. *Hypertension* 2007;50:1012–8.
10. Remais JV, Zeng G, Li G, Tian L, Engelgau MM. Convergence of non-communicable and infectious diseases in low-and middle-income countries. *International Journal of Epidemiology* 2013, 42(1):221-227.
11. Chang AY, Gómez-Olivé FX, Manne-Goehler J, Wade AN, Tollman S, Gaziano TA, Salomon JA. Multimorbidity and care for hypertension, diabetes and HIV among older adults in rural South Africa. *Bulletin of the World Health Organization* 2019, 97(1):10.
12. George S, McGrath N, Oni T. The association between a detectable HIV viral load and non-communicable diseases comorbidity in HIV positive adults on antiretroviral therapy in Western Cape, South Africa. *BMC Infectious Diseases* 2019, 19(1):1-11.
13. Patel P, Rose CE, Collins PY, *et al*. Noncommunicable diseases among HIV-infected persons in low-income and middle-income countries: a systematic review and meta-analysis. *AIDS* 2018;32 Suppl 1:S5–20.
14. Manne-Goehler J, Atun R, Stokes A, *et al*. Diabetes diagnosis and care in sub-Saharan Africa: pooled analysis of individual data from 12 countries. *Lancet Diabetes Endocrinology* 2016;4:903–12.

For peer review only - http://bmjopen.bmj.com/site/about/guidelines.xhtml

15. UNAIDS. *Fact Sheet- World AIDS Day 2021*. Available at: https://www.unaids.org/en/resources/fact-sheet (Accessed 16 February 2023)

16. Ford N, Ball A, Baggaley R, Vitoria M, Low-Beer D, Penazzato M, Vojnov L, Bertagnolio S, Habiyambere V, Doherty M. The WHO public health approach to HIV treatment and care: looking back and looking ahead. *The Lancet Infectious Diseases* 2018, 18(3):e76-e86.

17. Ciccacci F, Tolno VT, Doro Altan AM, Liotta G, Orlando S, Mancinelli S, Palombi L, Marazzi MC. Noncommunicable Diseases Burden and Risk Factors in a Cohort of HIV+ Elderly Patients in Malawi. *AIDS Research and Human Retroviruses.* 2019 35(11-12):1106-1111.

18. Achwoka D, Oyugi JO, Mutave R, Munywoki P, Achia T, Akolo M, Muriuki F, Muthui M, Kimani J: High prevalence of non-communicable diseases among key populations enrolled at a large HIV prevention & treatment program in Kenya. *PLoS One* 2020, 15(7):e0235606.

19. Achwoka D, Waruru A, Chen TH, Masamaro K, Ngugi E, Kimani M, Mukui I, Oyugi JO, Mutave R, Achia T *et al*: Noncommunicable disease burden among HIV patients in care: a national retrospective longitudinal analysis of HIV-treatment outcomes in Kenya, 2003-2013. *BMC Public Health* 2019, 19(1):372.

20. Kamkuemah, M., Gausi, B. & Oni, T. Missed opportunities for NCD multimorbidity prevention in adolescents and youth living with HIV in urban South Africa. *BMC Public Health* 2020; 20, 821.

21. Shiri T, Birungi J, Garrib AV, Kivuyo SL, Namakoola I, Mghamba J, Musinguzi J, Kimaro G, Mutungi G, Nyirenda MJ. Patient and health provider costs of integrated HIV, diabetes and hypertension ambulatory health services in low-income settings—an empirical socio-economic cohort study in Tanzania and Uganda. *BMC Medicine* 2021, 19(1):1-15.

22. Jaffar S, Ramaiya K, Karekezi C, Sewankambo N, Katahoire AR, Kraef C, Mutungi G, Musinguzi J, Birungi J, Garrib A. Controlling diabetes and hypertension in sub-Saharan Africa: lessons from HIV programmes. *The Lancet* 2021, 398(10306):1111-1113.

23. Haregu TN, Setswe G, Elliott J, Oldenburg B. Integration of HIV/AIDS and noncommunicable diseases in developing countries: rationale, policies and models. *International Journal of Healthcare* 2015, 1(1):21.

For peer review only - http://bmjopen.bmj.com/site/about/guidelines.xhtml

24. Guwatudde D, Nankya-Mutyoba J, Kalyesubula R, Laurence C, Adebamowo C, Ajayi I, Bajunirwe F, Njelekela M, Chiwanga FS, Reid T. The burden of hypertension in sub-Saharan Africa: a four-country cross sectional study. *BMC Public Health* 2015, 15(1):1-8.

25. Jaffar S, Gill G. The crisis of diabetes in sub-Saharan Africa. *Lancet Diabetes and Endocrinology* 2017, 5(8):574-575.

26. Jaffar S. Diabetes and other non-communicable diseases in Africa: a potential disaster in the waiting. *Lancet Diabetes & Endocrinology* 2016, 4(11):875-877.

27. Price AJ, Crampin AC, Amberbir A, Kayuni-Chihana N, Musicha C, Tafatatha T, Branson K, Lawlor DA, Mwaiyeghele E, Nkhwazi L. Prevalence of obesity, hypertension, and diabetes, and cascade of care in sub-Saharan Africa: a cross-sectional, population-based study in rural and urban Malawi. *Lancet Diabetes & Endocrinology* 2018, 6(3):208-222.

28. Ameh S, Klipstein-Grobusch K, D'ambruoso L, Kahn K, Tollman SM, Gómez-Olivé FX. Quality of integrated chronic disease care in rural South Africa: user and provider perspectives. *Health Policy and Planning* 2017, 32(2):257-266.

29. Birungi J, Kivuyo S, Garrib A, Mugenyi L, Mutungi G, Namakoola I, Mghamba J, Ramaiya K, Wang D, Maongezi S. Integrating health services for HIV infection, diabetes and hypertension in sub-Saharan Africa: a cohort study. *BMJ Open* 2021, 11(11):e053412.

30. Divala OH, Amberbir A, Ismail Z, Beyene T, Garone D, Pfaff C, Singano V, Akello H, Joshua M, Nyirenda MJ. The burden of hypertension, diabetes mellitus, and cardiovascular risk factors among adult Malawians in HIV care: consequences for integrated services. *BMC Public Health* 2016, 16(1):1-11.

31. Duffy M, Ojikutu B, Andrian S, Sohng E, Minior T, Hirschhorn LR. Non-communicable diseases and HIV care and treatment: models of integrated service delivery. *Tropical Medicine & International Health* 2017, 22(8):926-937.

32. Golovaty I, Sharma M, Van Heerden A, van Rooyen H, Baeten JM, Celum C, Barnabas RV. Cost of integrating non-communicable disease screening into home-based HIV testing and counseling in South Africa. *Journal of Acquired Immune Deficiency Syndromes* 2018, 78(5):522.

For peer review only

For peer review only - http://bmjopen.bmj.com/site/about/guidelines.xhtml

33. Iwelunmor J, Ezechi O, Obiezu-Umeh C, Gbajabiamila T, Musa AZ, Oladele D, Idigbe I, Ohihoin A, Gyamfi J, Aifah A. Capabilities, opportunities and motivations for integrating evidence-based strategy for hypertension control into HIV clinics in Southwest Nigeria. *PloS One* 2019, 14(6):e0217703.

34. Fairall LR, Folb N, Timmerman V, Lombard C, Steyn K, Bachmann MO, Bateman ED, Lund C, Cornick R, Faris G. Educational outreach with an integrated clinical tool for nurse-led non-communicable chronic disease management in primary care in South Africa: a pragmatic cluster randomised controlled trial. *PLoS Medicine* 2016, 13(11):e1002178.

35. Kwarisiima D, Atukunda M, Owaraganise A, Chamie G, Clark T, Kabami J, Jain V, Byonanebye D, Mwangwa F, Balzer LB. Hypertension control in integrated HIV and chronic disease clinics in Uganda in the SEARCH study. *BMC Public Health* 2019, 19(1):1-10.

36. Masupe T, Mashalla Y, Seloilwe E, Jibril H, Medhin H. Integrated management of HIV/NCDs: knowledge, attitudes, and practices of health care workers in Gaborone, Botswana. *African Health Sciences* 2019, 19(3):2312-2323.

37. Some D, Edwards JK, Reid T, Van den Bergh R, Kosgei RJ, Wilkinson E, Baruani B, Kizito W, Khabala K, Shah S. Task shifting the management of non-communicable diseases to nurses in Kibera, Kenya: does it work? *PLoS One* 2016, 11(1):e0145634.

38. Bukenya, D., Van Hout, MC ., Shayo, E., Kitabye, I., Musenze, B., Kasidi, J.R., Jaffar, S., Seeley, J. Accessing Integrated Health Care Services for HIV, Diabetes Mellitus and Hypertension in Selected Health Facilities in Kampala and Wakiso Districts, Uganda. *PLOS Global Health*. 2021; Early Online . DOI: https://doi.org/10.1371/journal.pgph.0000084

39. Van Hout, MC., Zalwango, F., Akugizibwe, M., Namulundu Chaka, M., Bigland, C., Birungi, J., Jaffar, S., Bachmann, M., Murdoch, J. (2022). Women living with HIV, diabetes and/or hypertension multimorbidity in Uganda: A qualitative exploration of experiences accessing a *'one stop'* integrated care service. *Journal of Integrated Care.* 2022, Early Online. https://www.emerald.com/insight/1476-9018.htm

40. Akugizibwe, M., Zalwango, F., Namulundu Chaka, M., Namakoola, I., Birungi, J., Okebe, J., Bachmann, M., Murdoch J., Jaffar, S., Van Hout, MC. "After all, we are all sick" : Multi-stakeholder understandings of stigma associated with integrated management of HIV, Diabetes and Hypertension

For peer review only - http://bmjopen.bmj.com/site/about/guidelines.xhtml

at selected government clinics in Uganda. *BMC Health Services Research.* 2023, 23(20). https://doi.org/10.1186/s12913-022-08959-3

41. Shayo, E., Kivuyo, S., Seeley, J., Bukenya, D., Karoli, P., Mfinanga, S., Jaffar, S., Van Hout, MC. The acceptability of integrated healthcare services for HIV and non-communicable diseases: Experiences from patients and healthcare workers in Tanzania. *BMC Health Services Research* 2022, 22, 655 https://doi.org/10.1186/s12913-022-08065-4

42. Vorkoper S, Kupfer LE, Anand N, Patel P, Beecroft B, Tierney WM, Ferris R, El-Sadr WM: Building on the HIV chronic care platform to address noncommunicable diseases in sub-Saharan Africa: a research agenda. *AIDS (London, England)* 2018, 32(Suppl 1):S107.

43. Bonghaseh, DT., Bigland, C., Van Hout, MC. Understanding knowledge, attitudes, perceptions and practices towards cardiovascular disease risk and prevention in patients living with HIV in Sub-Saharan Africa: A synthesis of qualitative literature. *Journal of Sustainable Development Africa* 2022; 24(2):11-29.

44. Adeyemi O, Lyons M, Njim T, Okebe J, Birungi J, Nana K, Claude Mbanya J, Mfinanga S, Ramaiya K, Jaffar S *et al*: Integration of non-communicable disease and HIV/AIDS management: a review of healthcare policies and plans in East Africa. *BMJ Global Health* 2021, 6(5).

45. Juma K, Reid M, Roy M, Vorkoper S, Temu TM, Levitt NS, Oladepo O, Zakus D, Yonga G. From HIV prevention to non-communicable disease health promotion efforts in sub-Saharan Africa: a narrative review. *Aids* 2018, 32:S63-S73.

46. Lupafya PC, Mwagomba BLM, Hosig K, Maseko LM, Chimbali H. Implementation of policies and strategies for control of noncommunicable diseases in Malawi: challenges and opportunities. *Health Education & Behavior* 2016, 43(1_suppl):64S-69S.

47. Shayo, E., Garrib, A., Birungi, J., Kivuyo, S. Mfinanga, S., Nyirenda, M., Namakola, I., Ramaiya, K., Bachmann, M., Cullen, W., Lazarus, J., Gill, G., Van Hout, MC., Shiri, T., Bukenya, D., Snell, H., Nanfuka, M., Cuevas, L., Shimwela, M., Mutungi, G., Musinguzi, J., Mghamba, J., Mangozi, S., Mugisha, K., Jaffar, S., Smith, P., Sewankamo N. Ethical issues in intervention studies on the management of treatment of diabetes and hypertension in sub Saharan Africa. *BMJ Global Health.* 2020, 5(7), pp e002193.

For peer review only - http://bmjopen.bmj.com/site/about/guidelines.xhtml

48. Murtagh, S., McCombe, G., Bachmann, M., Garrib, A., Jaffar, -S-., Lazarus, J., Mfinanga, S., Ramaiya, K., Sewankamo, N., Van Hout, MC., Cullen, W. (2021). Integrating diabetes, hypertension and HIV care in Africa: Delphi consensus study on international best practice *BMC Health Services Research* 2021; 21, 1235. Early Online https://doi.org/10.1186/s12913-021-07073-0

49. McCombe, G., Lim, J., Van Hout, MC., Lazarus, J., Bachmann, M., Garrib, A., Jaffar, S., Mfinanga, S., Ramaiya, K., Sewankamo, N., Cullen, W. Integrating care for diabetes and hypertension with HIV care in sub-Saharan Africa: A scoping review. *International Journal of Integrated Care.* 2021; 22(1): 6, 1–22. DOI: https://doi.org/10.5334/ijic.5839

50. Singh S, Kirk O, Jaffar S, Karakezi C, Ramaiya K, Kallestrup P, Kraef C. Patient perspectives on integrated healthcare for HIV, hypertension and type 2 diabetes: a scoping review. *BMJ Open* 2021, 11(11):e054629.

51. Haldane V, Legido-Quigley H, Chuah FLH, Sigfrid L, Murphy G, Ong SE, Cervero-Liceras F, Watt N, Balabanova D, Hogarth S. Integrating cardiovascular diseases, hypertension, and diabetes with HIV services: a systematic review. *AIDS Care* 2018, 30(1):103-115.

52. Moyo M, Musekiwa A. Protocol for updated systematic review and meta-analysis on the burden of non-communicable diseases among people living with HIV in sub-Saharan Africa. *BMJ Open* 2022;12(5):e055895.

53. Kintu A, Sando D, Okello S, Mutungi G, Guwatudde D, Menzies NA, Danaei G, Verguet S. Integrating care for non-communicable diseases into routine HIV services: key considerations for policy design in sub-Saharan Africa. *Journal of the International AIDS Society* 2020, 23:e25508.

54. Mahomed OH, Asmall S. Development and implementation of an integrated chronic disease model in South Africa: lessons in the management of change through improving the quality of clinical practice. *International Journal of Integrated Care* 2015, 15.

55. Pfaff C, Scott V, Hoffman R, Mwagomba B. You can treat my HIV–But can you treat my blood pressure? Availability of integrated HIV and noncommunicable disease care in northern Malawi. *African Journal of Primary Health Care and Family Medicine* 2017, 9(1):1-8.

56. Rawat A, Uebel K, Moore D, Yassi A. Integrated HIV-care into primary health care clinics and the influence on diabetes and hypertension care: an interrupted time series analysis in Free State, South

For peer review only - http://bmjopen.bmj.com/site/about/guidelines.xhtml

Africa over 4 years. *Journal of Acquired Immune Deficiency Syndromes* 2018, 77(5):476-483.

57. Mwagomba BLM, Ameh S, Bongomin P, Juma PA, MacKenzie RK, Kyobutungi C, Lukhele N, Mwangi KJM, Amberbir A, Klipstein-Grobusch K. Opportunities and challenges for evidence-informed HIV-noncommunicable disease integrated care policies and programs: lessons from Malawi, South Africa, Swaziland and Kenya. *AIDS* 2018, 32:S21-S32.
58. Fisher, E.B., Boothroyd, R.I., Elstad, E.A. *et al.* Peer support of complex health behaviors in prevention and disease management with special reference to diabetes: systematic reviews. *Clinical Diabetes and Endocrinology* 2017; 3, 4.
59. Egbujie BA, Delobelle PA, Levitt N, Puoane T, Sanders D, van Wyk B. Role of community health workers in type 2 diabetes mellitus self-management: a scoping review. *PLoS One.* 2018;13(6):e0198424.
60. Decroo T, Telfer B, Dores CD*, et al* Effect of Community ART Groups on retention-in-care among patients on ART in Tete Province, Mozambique: a cohort study *BMJ Open* 2017;7:e016800.
61. Hagey JM, Li X, Barr-Walker J, Penner J, Kadima J, Oyaro P, Cohen CR. Differentiated HIV care in sub-Saharan Africa: a scoping review to inform antiretroviral therapy provision for stable HIV-infected individuals in Kenya. *AIDS Care*. 2018 Dec;30(12):1477-1487.
62. Jaffar S, Amuron B, Foster S, Birungi J, Levin J, Namara G, Nabiryo C, Ndembi N, Kyomuhangi R, Opio A, Bunnell R, Tappero JW, Mermin J, Coutinho A, Grosskurth H; Jinja trial team. Rates of virological failure in patients treated in a home-based versus a facility-based HIV-care model in Jinja, southeast Uganda: a cluster-randomised equivalence trial. *Lancet.* 2009; 374(9707):2080-2089.
63. Van Hout M-C, Bachmann M, Lazarus JV, Shayo EH, Bukenya D, Picchio CA, Nyirenda M, Mfinanga SG, Birungi J, Okebe J.Strengthening integration of chronic care in Africa: protocol for the qualitative process evaluation of integrated HIV, diabetes and hypertension care in a cluster randomised controlled trial in Tanzania and Uganda. *BMJ Open* 2020, 10(10):e039237.
64. Mfinanga SG, Nyirenda MJ, Mutungi G, Mghamba J, Maongezi S, Musinguzi J, Okebe J, Kivuyo S, Birungi J, Van Widenfelt E. Integrating HIV, diabetes and hypertension services in Africa: study protocol for a cluster randomised trial in Tanzania and Uganda. *BMJ Open* 2021, 11(10):e047979.
65. Georgeu D, Colvin CJ, Lewin S, *et al*. Implementing nurse-initiated and managed antiretroviral

For peer review only - http://bmjopen.bmj.com/site/about/guidelines.xhtml

treatment (NIMART) in South Africa: a qualitative process evaluation of the stretch trial. *Implementation Science* 2012;7:66.

66. Craig P, Dieppe P, Macintyre S, *et al*. Developing and evaluating complex interventions: the new medical Research Council guidance. *BMJ* 2008;337:a1655.
67. Moore G, Audrey S, Barker M, *et al*. Process evaluation in complex public health intervention studies. London: MRC Population Health Science Research Network 2014.
68. Linnan L, Steckler A. Process evaluation for public health interventions and research. An overview. In: Steckler A, Linnan L,eds. *Process evaluation for public health interventions and research*. 1 edn. San Francisco: Josset-Bass, 2002: 1–23.
69. Murdoch J, Curran R, Bachmann M, *et al*. Strengthening the quality of paediatric primary care: protocol for the process evaluation of a health systems intervention in South Africa. *BMJ Global Health* 2018;3:e000945.
70. Bronfenbrenner U. Toward an experimental ecology of human development. *Am Psychol* 1977;32:513–31.
71. Bronfenbrenner U. *The ecology of human development: experiments by nature and design.* Cambridge, MA: Harvard University Press, 1979.
72. Hawe P, Shiell A, Riley T. Theorising interventions as events in systems. *Am J Community Psychol.*2009;43(3-4):267-76.
73. Murdoch, J., Curran, R., Cornick, R. *et al.* Addressing the quality and scope of paediatric primary care in South Africa: evaluating contextual impacts of the introduction of the Practical Approach to Care Kit for children (PACK Child). *BMC Health Services Research* 2020, 20, 479.
74. Meghani A, Ssemugabo C, Pariyo G, Hyder AA, Rutebemberwa E, Gibson DG: Curbing the rise of noncommunicable diseases in Uganda: perspectives of policy actors. *Global Health: Science and Practice* 2021, 9(1):149-159.
75. Katende D, Mutungi G, Baisley K, Biraro S, Ikoona E, Peck R, Smeeth L, Hayes R, Munderi P, Grosskurth H. Readiness of Ugandan health services for the management of outpatients with chronic diseases. *Tropical Medicine & International Health* 2015, 20(10):1385-1395.

For peer review only - http://bmjopen.bmj.com/site/about/guidelines.xhtml

76. Grimshaw JM, Zwarenstein M, Tetroe JM, *et al*. Looking inside the black box: a theory-based process evaluation alongside a randomised controlled trial of printed educational materials (theOntario printed educational message, OPEM) to improve referral and prescribing practices in primary care in Ontario, Canada. *Implementation Science* 2007;2:38.

77. Karlsson G. *Psychological qualitative research from a phenomenological perspective*. Almqvist & Wiksell International,1993.

78. Mitchell CJ. Typicality and the case study. In: Ellen PF, editor. *Ethnographic research: a guide to general conduct.* New York: Academic 1984:238-41.

For peer review only

For peer review only - http://bmjopen.bmj.com/site/about/guidelines.xhtml

Table 1: Profile of Tanzania and Uganda regarding prevalence of three chronic conditions (hypertension, diabetes and HIV)

Indicators	Uganda	Tanzania
Income levels	Low	Middle
Population size	49,332,360	65,497,748
HIV prevalence	6.2%	4.9%
Diabetes prevalence	4.6%	9.1%,
Hypertension prevalence	33.4%	26.0%
Doctors density/100 000 population	0.1	3

For peer review only

Table 2 Objectives, contextual features and data collection methods of INTE-COMM process evaluation

**Objective/research question 1:** *What are patients, health care providers' and lay health workers' perceptions and experiences of integrated management of HIV, Diabetes, and Hypertension care in the community and at the health facility level?*		
**Contextual level/feature**	**Data Collection Method**	**Sources of information**
**Meso** Time and resources required; utilisation of policies and protocols; organisation and process of monthly meetings; training, organisation and work allocation of nurse and lay health worker; implementation processes and adaptations to embed intervention within communities<br>**Micro** Individual patient pathways to integrated care; relationship between intervention and management of HTN, DM and HIV within everyday life	Interview	Service users (patients stable on treatment)
	Interview	Health care providers (health facility managers, physicians/clinician, nurses, lay health workers, patient club facilitators)
	Focus groups	Community members/ leaders (8-12 participants)per group
	Interview	Representatives from Patient clubs/groups/peer educators.
	Interview	INTE-COMM investigators
**Objective/research question 2:** *What key contextual and process factors influence patients' and healthcare providers' uptake and acceptability of integrated management of HIV, Diabetes, and Hypertension care in the community and at the health facility level?*		
**Macro** Wider cultural discourses of HIV, hypertension and diabetes (e.g. HIV stigma); availability of medicines; socio-economic, trained staff, structures where services are delivered<br>**Meso** Geographic and demographic profile of communities and facilities; medicines supply; workforce arrangements of main social actors involved in intervention delivery (i.e. nurses and lay workers, patients, carers, community leaders); local practices and protocols; patient access and flow through community; referral pathways; role of traditional healers; and the different sites and scenes which impact on how INTE-COMM is adapted and delivered (e.g. local area management meetings)<br>**Micro**-individual level: influence of privacy and confidentiality in accessing care, trust placed to those delivering the intervention, mobility factor, economic factors.	Interview	Policymakers and Reps of NGOs (ministerial policymakers and provincial/regional/district level clinical/health senior management (Directors/ Programme managers/ Commissioners, Assistant Commissioner of NCDs; Commissioner/Programme manager of the National AIDS Control programme)
	Interview	Health care providers (health facility managers, physicians/clinicians, nurses, lay health workers, patient club facilitators) peer groups/educators, traditional healers.<br>Service care users (patients stable on treatment)
	Focus groups	Community members/leaders (8-12 participants per group)
**Objective/research question 3:** *To evaluate implementation and theoretical fidelity of the intervention*		
**Meso** Main (i.e. monthly meetings, interactions at dispensaries) activities and subsidiary activities (e.g. additional training/meetings, receptionist screening of patients) of intervention delivery; and interactional arrangements of each activity: structural arrangement at the point of service delivery (friendly environment, privacy and confidentiality issues)	Non-participant observations with structured field notes.	Service delivery processes, the flow of clinical procedures, the nature of services given, listening in patients' and health workers' conversations; freedom in patients entering the service point interactions, discussing and asking questions when waiting for the services
**Objective/research question 4:** *What factors can potentially influence the scaling up of integrated management of HIV, Diabetes, and Hypertension at the community and health facility level?*		
**cro** Cultural and structural characteristics, networks and communication, and external policies and incentives<br>**Meso** Community and facility resource constraints; drug supply and patient medicine clubs; referral pathways	Interview	Service users
	Interview	Healthcare providers (health facility managers, physicians/clinicians, nurses, lay health workers, patient club facilitators)
	Interview	Representatives from Patient clubs/group/peer educators
	Focus Groups	Community leaders and community members (8-12 participants per group)
	Interview	Policymakers and Reps of NGOs (ministerial policymakers and provincial/regional/district level clinical/health senior management (Directors/ Programme managers/ Commissioners, Assistant Commissioner of NCDs; Commissioner/Programme manager of the National AIDS Control programme)

For peer review only

For peer review only - http://bmjopen.bmj.com/site/about/guidelines.xhtml

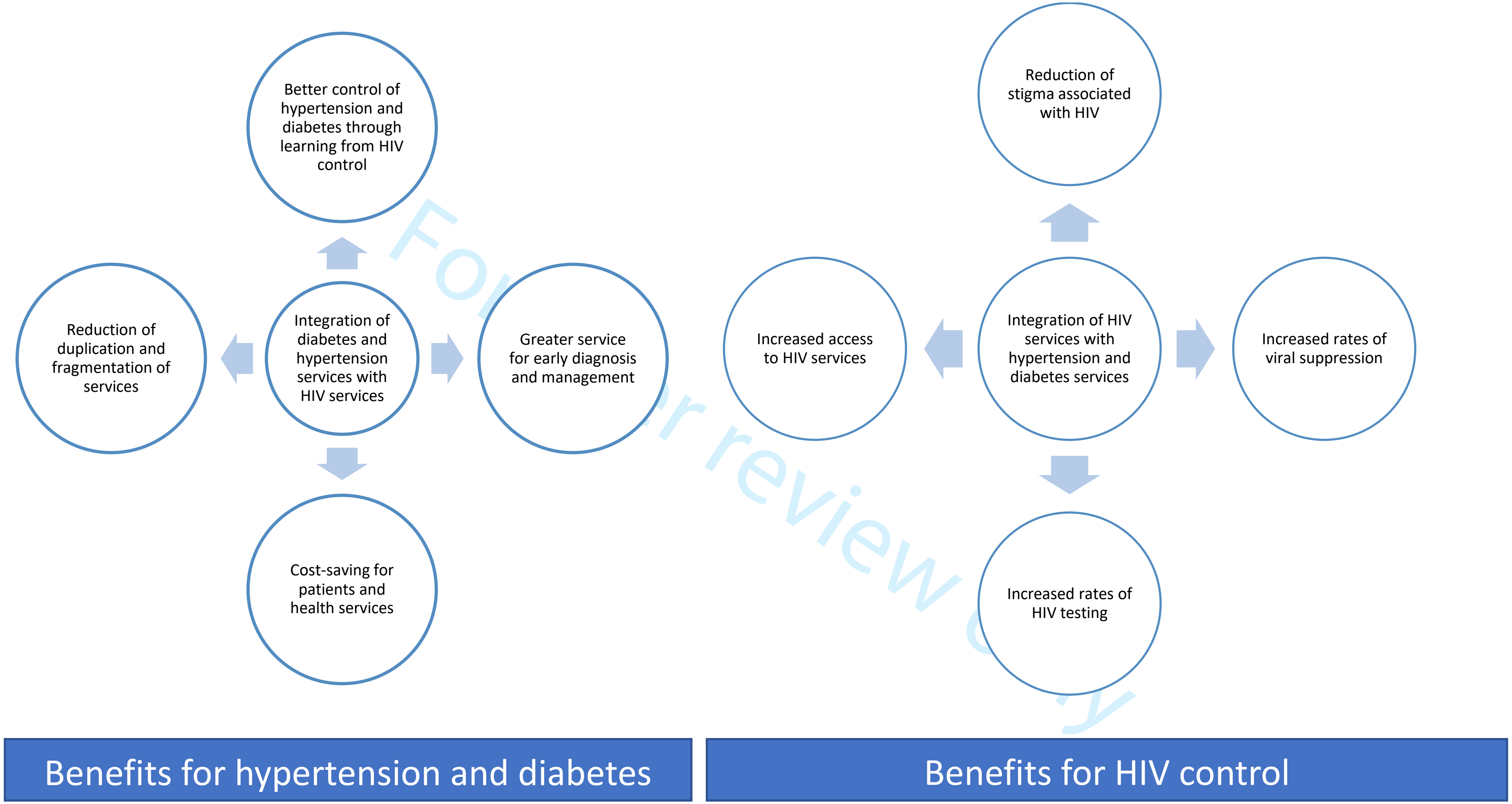

Figure 1: Benefits of integrated HIV, hypertension and diabetes care in general

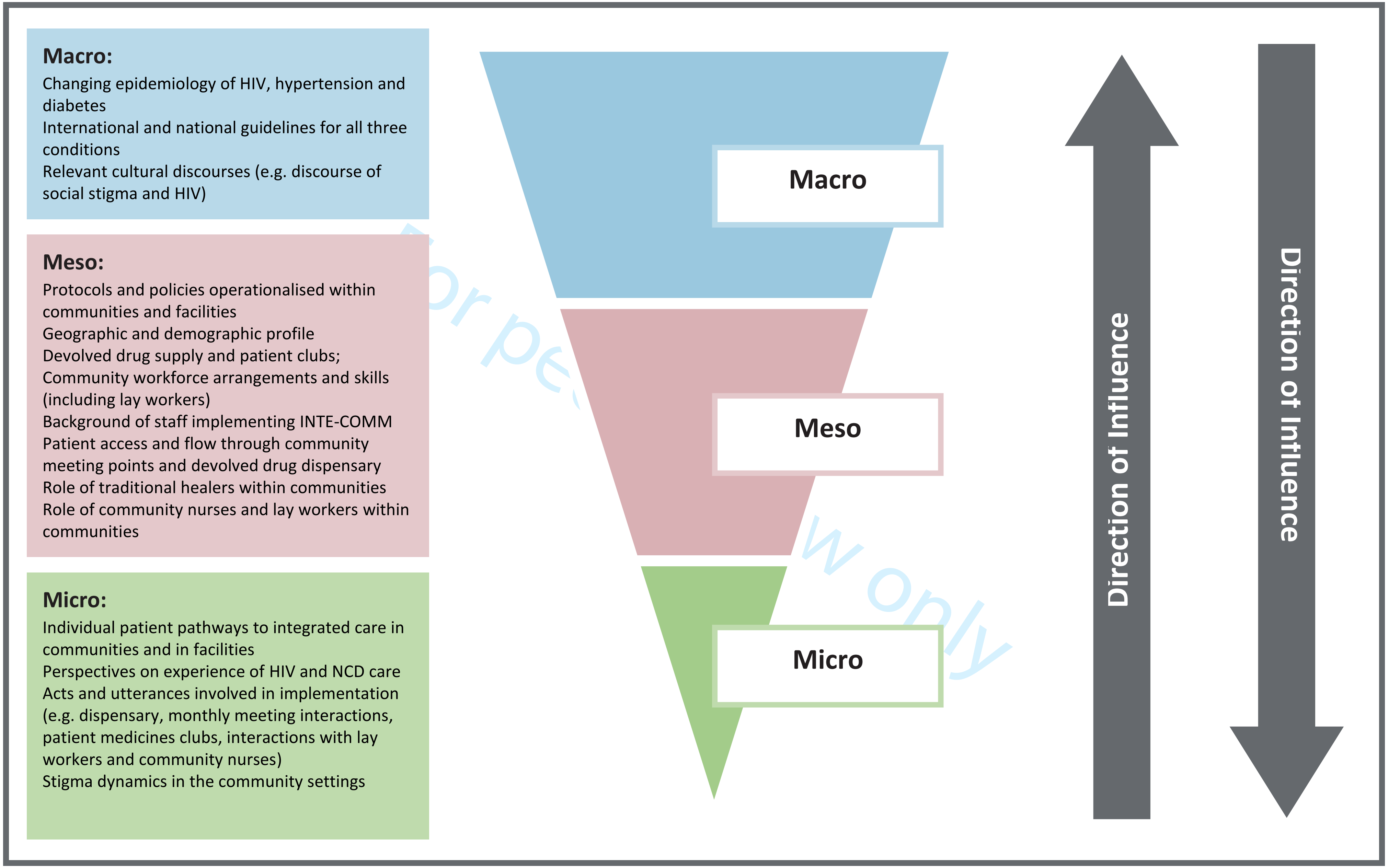

Figure 2: Potential contextual influences on INTE-COMM programme implementation cascade

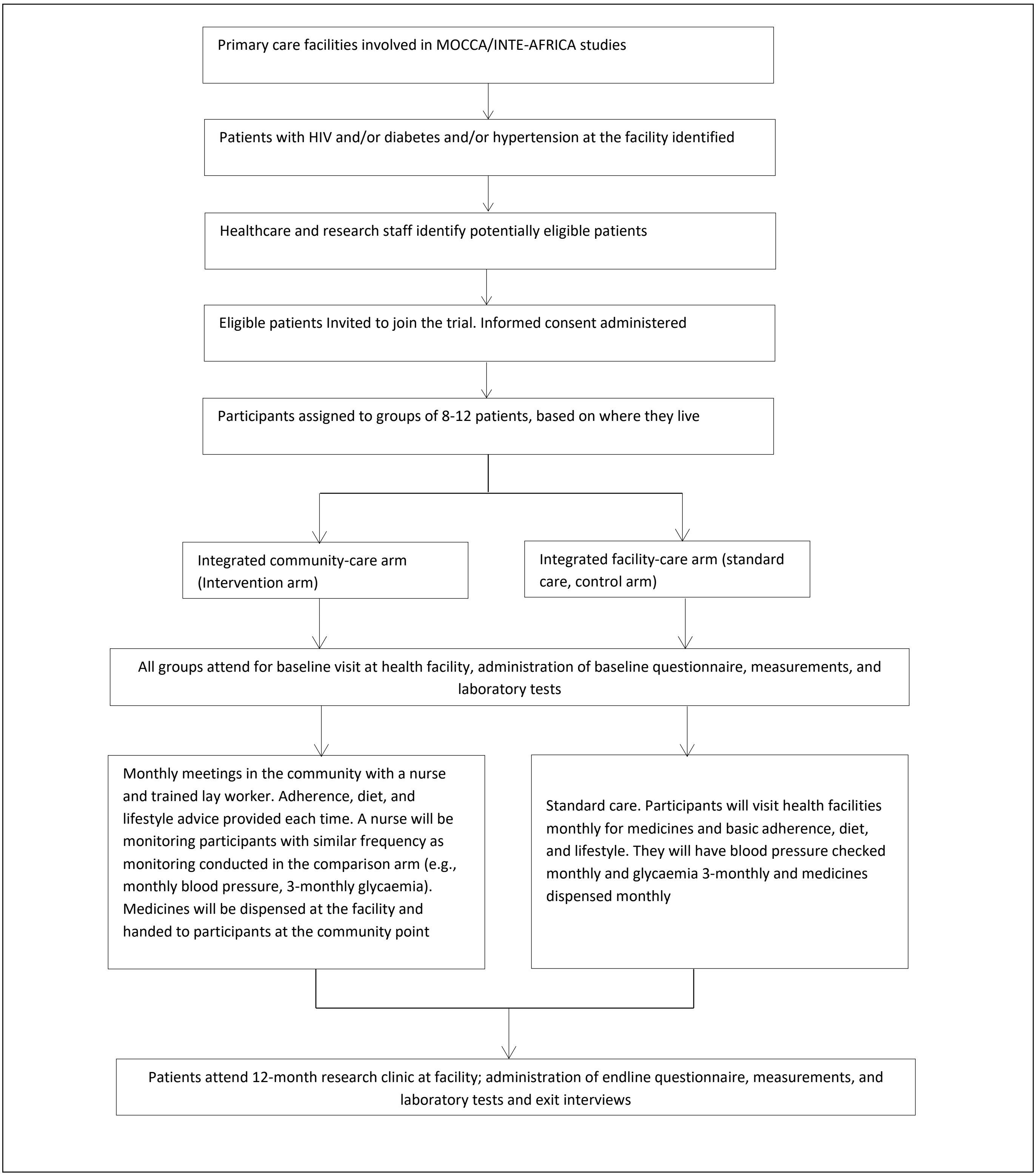

Figure 3. INTE-COMM study schema.

Supplemental File: Data Collection Guides

Health care users/patients (interview guide)

Introduction

Thank you for consenting to participate in this study. As mentioned to you earlier, this is a collaborative research project involving Ministry of Health, Medical Research Council/Uganda Virus Research Institute/ National Institute for Medical Research-Tanzania and London School of Hygiene and Tropical Medicine Research Unit, University College London and the Liverpool School of Tropical Medicine in UK. The aim of the research is to understand your perspectives as service users towards the management of the chronic disease conditions (HIV, diabetes and hypertension or combination of these) in relation to the integrated care model when health services for these conditions are provided together. Participation is purely voluntary, and refusal does not affect the quality of care that you will receive. We assure you of the confidentiality of the information that you will provide. We request you to allow us to use the tape recorder just to ensure the correctness of the information when writing the report.

Socio-demographic and treatment history

1. Briefly tell me about yourself. *Probe for: age, marital status, education level, source of livelihood, religion, If they have children (how many and ages)*
2. Please tell me about the medical conditions that you are suffering from? *(Probe: diabetes, hypertension, HIV, combination of conditions);*

   -When did you learn that you have this/these conditions?

   -How did you come to know about the condition(s)? *Probe for: Symptoms*

   -What actions did you initially take after learning/knowing that you have this/these conditions? *(Probe for: Self-medication, consultation with health facilities, spiritual healers, took/used herbal medicine prayed etc.)* -And reasons for taking the above action?

   - Whether the options were sought concurrently or simultaneously and why?
3. What do you think are the main causes of the disease that you are suffering from? *Probe for: what are the local/cultural beliefs attached to the cause of the disease(s)?* Probe for information about healthy diet/exercise behaviours
4. How do you think the above-mentioned disease condition can be prevented? *probe for: what are the local/cultural beliefs attached to the cause of the disease(s)?*
5. How do you think patients living with the above mentioned diseases can be better managed?

For peer review only

Perceptions on INTE-COMM

6. What do you know about the integrated community-based care of HTN, DM, and HIV (INTE-COMM)? *Probe for:*

   -Knowledge about INTE-COMM, ability to differentiate INTE-COMM from facility based integration, source of this information, etc.

7. What are your views/perceptions on the integrated community-based care model? *Probe for:*

   *-**Willingness/unwillingness** of the patients to seek and continue seeking care in such a decentralized integrated model and reasons why*?

   - ***Possible positives** about integration e.g. time spent, perceived quality of care, stigma, costs, continuity of care, medicines availability, perceptions of healthcare provider expertise, follow-up support, Interpersonal relationships, etc.)*
   - ***Possible negatives** about integration e.g. time spent at the facility, perceived quality of care, stigma, costs, continuity of care, medicines availability, perceptions of healthcare provider expertise, follow-up support, etc.) Probe for childcare responsibilities if applicable*

8. What services do you think should be integrated and delivered with this model? *Probe for: Diagnostic, medication, consultation, health education sessions or all, etc.*) and the reasons why? Probe for maternity services

9. What services do you think should not be integrated and delivered with this model? *Probe for: Diagnostic, medication, consultation, health education sessions or all, etc.*) *and the reasons why?*

Contextual factors

10. Who are the most trusted people by patients in the community to deliver drug refills, screening, and provide health education and where is the focal point and why?

11. Which category of patients are likely to embrace INTE-COMM easily and why? *Probe for: patients in rural vs Urban, educated vs uneducated, men vs women, rich vs poor, young vs aging, those who subscribe to patient clubs' vs none subscribers, etc.*

12. Which categories of patients are likely not to embrace INTE-COMM easily and why? *Probe for: patients in rural vs Urban, educated vs uneducated, men vs women, rich vs poor, young vs aging, those who subscribe to patient clubs' vs none subscribers, etc.*

For peer review only

13. What factors can enable/facilitate patients' easy access to services provided under INTECOMM? *Probe for: Individual, community, and health facility level facilitating factors*
14. What factors make it hard/hinder patients from accessing the services provided under INTE-COMM? *Probe for: Individual, community, and health facility level barriers*. Probe for family responsibilities
15. What do you recommend should be done to enable patients' easy access to services provided under INTE-COMM?
16. What factors do you think enable/facilitate effective/optimal delivery of the community based integrated care model in Uganda? *Probe for: individual-level factors, community level including cultural factors, health systems level, policy level, and political factors, etc.*
17. What factors do you think hinder the effective/optimal delivery of the community-based integrated care model in Uganda? *Probe for*: *individual-level factors, community level including cultural factors, health systems level, and political factors.*
18. What do you recommend should be done to ensure effective delivery of INTE-COMM?
19. Do you think a community-based integrated care model can be sustainable in Uganda? *Probe for*:

    -Factors that can enable the delivery of a sustainable community-based integrated service.

    -Factors that can hinder the delivery of a sustainable community-based integrated service?

Healthcare-seeking experiences of integrated care services

20. Now that you are receiving medical treatment from the health facility/community post, What enables/motivates you to continue seeking health care services for the conditions that you have? *Probe for interpersonal relationships, waiting time, distance to reach the clinic, availability of medicine and supplies, etc.)*
21. What demotivates you in seeking health care services from a health facility/community post for the condition(s) that you have? *Probe for: Interpersonal relationships, waiting time, distance to reach the clinic, availability of medicine and supplies, etc.)* Probe for family responsibilities, childcare, finances
22. What motivates you to comply with the medical treatment provided by the health workers from the health facility/community post for the condition(s) you have?
23. What demotivates you to comply with the medical treatment provided by the health workers from the health facility/community post for the condition(s) you have?

For peer review only

24. For the patients who refused, or dropped out from this intervention(INTE-COOM) what do you think were their fears?
25. How do you suggest should be done to help address these fears in order to have more patients join and benefit from this intervention?

Process factors

26. Kindly tell me about the procedures that you normally follow when you come to receive the services. *Probe for:*

-How easy is it for you to follow and comply with those stipulated procedures? -How difficult is it for you to follow and comply with those stipulated procedures (probe about stigma, shyness, lack of clarity, etc.)?

-How do you suggest these procedures should be improved?

27. Tell me about the quality of services that you receive from the health facility/community based integrated care model. *Probe for*: *Interpersonal relationships, waiting time, distance to reach the clinic, availability of medicine and supplies, etc.)*

28. Which services were delivered well and why? *Probe for: health workers' knowledge, competence, availability of medical supplies, the attitude of health workers, inconsistencies in methods of delivery, evidence of patients' outcomes(impact), transport issues, etc.*

29. From the time you joined INTE-COMM, have you noticed any changes in your life? *Probe for: physical changes, economic, psychological, etc.)*

30. Which services were not delivered well and why? *Probe for: health workers' knowledge, competence, availability of medical supplies, the attitude of health workers, inconsistencies in methods of delivery, evidence of patients' outcomes, transport issues etc*

31. What are the recommended best practices for delivering effective community-based integrated care management for HIV, diabetes, and hypertension in Uganda?

Scalability of INTE-COMM

32. What are the factors that can enable/facilitate scaling up a community-based integrated care management of HIV, diabetes, and hypertension in Uganda?
33. What are the factors that can hinder the scaling up of a community-based integrated care management of HIV, diabetes, and hypertension in Uganda?
34. What do you recommend to be done to ensure an effective scaling up of INTE-COMM to other areas in the country? *Probe for: what recommendations, and by whom?*
35. Do you have any other comments in regard to this topic?

Thank you for your participation

For peer review only

Healthcare providers (Interview guide) Introduction

Thank you for consenting to participate in this study. This is a collaborative research project involving Ministry of Health, Medical Research Council/Uganda Virus Research Institute/National Institute for Medical Research-Tanzania and London School of Hygiene and Tropical Medicine Research Unit, University College London and the Liverpool School of Tropical Medicine in UK. In this study we are interested in understanding your reflection towards the health care service integrated model in the management of HV and NCD disease conditions. We have selected some few implementers directly working closely with patients seeking NCD/HIV care at the clinics and the policy makers. You will not be paid for participating in this study but we encourage you to participate because the answers we get from you and several others will be analyzed to get the general picture of the health service delivery integrated model at the community point of care. We are kindly requesting you to take part, and we assure you of the confidentiality of the information that you will provide. We request you to allow me to use the tape recorder just to ensure the correctness of the information when writing the report.

Socio-demographics and chronic disease treatment history

1. First of all, I would like to get the information about you. *Probe for and record: age, marital status, occupation, educational level, title/position, roles in integrated care service delivery*
2. Based on your experience in the management of chronic conditions, what are the main chronic disease conditions that people are suffer from in the community? (*Probe for HIV, diabetes and hypertension*)
3. What are the common health care seeking practices for community members for HIV, DM and HT? and why (*probe on self-medication, consultation with traditional healers, health facilities, spiritual healers)*
4. What services do you offer for these chronic conditions? *Probe for: each condition (DM, HIV, and HTN)*

Perceptions on INTE-COMM

5. What do you know about the integrated community-based care of HTN, DM, and HIV (INTE-COMM)? *Probe for:*

-Knowledge about INTE-COMM, ability to differentiate INTE-COMM from facility-based integration, source of this information, etc.

-Which services are delivered, who delivers the services-diagnostic, medication, consultation, health education sessions or all

-Which services are not integrated and delivered with this model? Probe for: Diagnostic, medication, consultation, health education sessions or all, etc) and the reasons why?

6. What are your views/perceptions on the integrated community-based care model? *Probe for:*

   *-**Willingness/unwillingness** of the patients to seek and continue seeking care in such a decentralized integrated model and reasons why*?

   - ***Possible positives** about integration e.g. time spent, perceived quality of care, stigma, costs, continuity of care, medicines availability, perceptions of healthcare provider expertise, follow-up support, Interpersonal relationships, etc.)*
   - ***Possible negatives** about integration e.g. time spent at the facility, perceived quality of care, stigma, costs, continuity of care, medicines availability, perceptions of healthcare provider expertise, follow-up support, etc.)*

7. What services do you think should be integrated and delivered with this model? *Probe for: Diagnostic, medication, consultation, health education sessions or all, etc.*) *and the reasons why?*
8. What services do you think should not be integrated and delivered with this model? *Probe for: Diagnostic, medication, consultation, health education sessions or all, etc.*) *and the reasons why?*
9. What factors can enable patients to accept participating in the INTE-COMM? *Probe for: **individual**, **community,** and **facility-level** enablers to patients' participation*
10. What factors can hinder other patients from accepting to participate in the INTE-COMM? *Probe for: **individual**, **community,** and **facility-level** barriers to patients' participation*
11. What can be done to improve patients' participation in this intervention?

Experience in HIV and NCD management

12. Kindly share with me your experience of delivering INTE-COMM, how satisfied are you with working in the community-based integrated model? *Probe for the reasons why?*
13. What are the patients' attitudes towards community-based integration compared to facility based integration for the management, of HIV, diabetes, and hypertension? *Probe for: evidence of **positive** and **negative** attitudes.*
14. What services are provided at the facility/community post for HIV, diabetes and hypertension? ask for one condition at a time. *Probe for: Diagnostic, medication, consultation, health education sessions or all, etc. as per the level of health facility related to HIV, DM, and HT.*

15. At the health facility/community post level, what do you do to ensure HIV, DM, and HT care is user-friendly? *Probe for: patient-health worker relationship, patient-patient relationship, health education, etc.)*
16. Now that you are offering medical treatment from the health facility/community post, What keeps you motivated to continue delivering health care services for your patients with chronic conditions? *Probe for: interpersonal relationships, transportation, allowances, patients' attitudes, physical space, training, patients' response to treatment, availability of medicine and supplies, etc.)*
17. What demotivates you, as you offer health care services to patients with chronic disease from a health facility/community post? *Probe for: interpersonal relationships, transportation, allowances, patients' attitudes, physical space, training, patients' response to treatment, availability of medicine and supplies, etc.)*
18. For the patients who refused, or dropped out from this intervention(INTE-COOM) what do you think were their fears/reasons?
19. What do you suggest should be done to help address these fears in order to have more patients join and benefit from this intervention?
20. While offering health care services for patients with diabetes, hypertension, and HIV under INTE-COMM, what challenges do you face?
21. How can these challenges be addressed to ensure optimal delivery of an effective INTE-COMM?
22. What are the challenges faced by patients in accessing services through INTE-COMM? *Probe for: distance, drug availability, stigma, physical space/infrastructure, time spent, costs, etc.)*
23. How can these challenges be addressed to ensure optimal access to INTE-COMM services?

Process factors

24. Kindly tell me about the procedures that you normally follow to serve your patients when they come to receive the services. *Probe for: -How easy is it for the patients to follow and comply with those stipulated procedures?*

-How difficult is it for patients to follow and comply with those stipulated procedures (probe about stigma, shyness, lack of clarity, etc.)?

-How do you suggest these procedures should be improved?

25. Tell me about the quality of services that you offer to patients through a community-based integrated care model Probe for: Which services were delivered well and why? (*including health workers' knowledge, competence, availability of medical supplies, the attitude of*

For peer review only

health workers, inconsistencies in methods of delivery, evidence of patients' outcomes(impact), transport issues, etc.)

26. Which services were not delivered well and why? *Probe for: health workers' knowledge, competence, availability of medical supplies, the attitude of health workers, inconsistencies in methods of delivery, evidence of patients' outcomes, transport issues, etc*

27. What are the recommended best practices for delivering effective community-based integrated care management for HIV, diabetes, and hypertension in Uganda?

28. What are the factors that may hinder the delivery of effective community-based integrated care services for HIV, DM, and HTN? *Probe for: Individual level, community, or structural/health facility level factors.*

29. . What do you think the best practices are, in delivering effective management of the mentioned chronic disease conditions in the same clinic at the community level?

30. What do you think should be done going forward to enable a robust and sustainable community-based integrated service for HIV/NCD? and by whom?

Scalability of INTE-COMM

31. Based on your experience in health care delivery, what are your views on scaling up INTECOMM to the rest of the areas in the country? Probe for: *-If it is necessary to scale up or not?*

    -How can scaling up of INTE-COM be done/achieved?

32. What are the factors that can enable/facilitate scaling up a community-based integrated care management of HIV, diabetes, and hypertension in Uganda? *Probe for: available strengths and opportunities for scaling up INTE-COMM.*

33. What are the factors that can hinder the scaling up of a community-based integrated care management of HIV, diabetes, and hypertension in Uganda? *Probe for: available weaknesses, and threats to scaling up INTE-COMM.*

34. What do you recommend to be done to ensure an effective scaling up of INTE-COMM to other areas in the country? *Probe for: what recommendations, and by whom?*

35. Do you have any other comments in regard to this topic?

Thank you for your participation

For peer review only

Policy makers at Ministerial and provincial/regional/district level clinical/health senior management (Director for NCD, HIV and curative services)/ NGO and international organisations (interview guide)

Introduction

Thank you for consenting to participate in this study. This is a collaborative research project involving Ministry of Health, Medical Research Council/Uganda Virus Research Institute National Institute for Medical Research-Tanzania and London School of Hygiene and Tropical Medicine Research Unit, University College London and the Liverpool School of Tropical Medicine in UK. In this study we are interested in understanding your reflection towards the health care service integrated model in the management of HV and NCD disease conditions. We have selected some few implementers directly working closely with patients seeking NCD/HIV care at the clinics and the policy makers. You will not be paid for participating in this study but we encourage you to participate because the answers we get from you and several others will be analysed to get the general picture of the health service delivery integrated model at the community point of care. We are kindly requesting you to take part, and we assure you of the confidentiality of the information that you will provide. We request you to allow me to use the tape recorder just to ensure the correctness of the information when writing the report.

Demographic profile

1. First of all, I would like to get the information about you. *Probe for age, sex, marital status, occupation, educational level, title/position, roles*

Experience of HIV and NCD

2. Based on your experience in the health care system, what are the main chronic disease conditions that people are suffering from? (*Probe for HIV, diabetes and hypertension*).
3. For HIV, diabetes and hypertension, can you describe the burden of disease for each, and if co-/multi-morbid.
4. What can you comment on the care seeking practices for community members for HIV, DM and HT? and why (*probe on self-medication, consultation with traditional healers, health facilities, spiritual healers)*
5. At an institution/organisational level, what does the facility management/district/national do to ensure HIV, DM and HT care is user friendly? *Probe for; diagnostic, drugs availability, health worker availability, waiting area and time, sitting arrangements, health education sessions, encouraging patient-patients' relationship/associations* Probe for:

-*The possibility to allocate resources for NCDs management*

For peer review only

-*What changes have been implemented to address such burden?*

Perceptions/Views on INTE-COMM

6. What do you know/have you heard about the integrated model service delivery? what ideas come up in your mind as a leader (*probe for: what it is, who to provide it, expectation-positive and negative*)
7. What services do you think should be integrated? (*Probe: Diagnostic, medication, consultation, health education sessions or all etc*) and the reasons why?
8. What are your perceptions towards such integrated model? (*Probe of the willingness of the patients to continue seeking care in such integrated model, stigma, etc.*)
9. Possible positives and negatives about integration e.g. *time spent at the facility, perceived quality of care, stigma, costs, continuity of care, medicines availability, perceptions of healthcare provider expertise, follow up support etc*.)
10. What are the foreseen factors that can enable successful implementation of a decentralised integrated care model at community level?
11. What are the foreseen factors that can hinder a successful implementation of a decentralised integrated care model at community level?
12. Do you foresee any difficulties in providing an optimal scaling up of community based integrated service?
13. What can be done to help achieve an optimal scaling up of community based integrated services in Uganda?

Thank you for your participation

For peer review only

Community Leaders/Members (Focus group discussion guide) Introduction

Thank you for consenting to participate in this study. As mentioned to you earlier, this is a collaborative research project involving Ministry of Health, Medical Research Council/Uganda Virus Research Institute National Institute for Medical Research-Tanzania and London School of Hygiene and Tropical Medicine Research Unit, University College London and the Liverpool School of Tropical Medicine in UK.

The aim of the research is to understand the Community perspectives towards management of the chronic disease conditions (HIV, diabetes and hypertension or combination of these) in relation to the community based integrated care model. Participation is purely voluntary, and refusal will be treated professionally with no negative repercussion.

Your views will provide the general picture of community care seeking practices including barriers and facilitators, also get opinion on the integrated care service model for patients seeking services for NCD/HIV care at the community health care posts or facility based clinics. You will not be paid for participating in this discussion but we encourage you to participate because the answers we get from you and several others will be analyzed to get the general picture and areas for improvement in strengthening the integrated service delivery model. We assure you of the confidentiality of the information that you will provide. The discussion will last between one to two hours depending on the richness of the information that you have. We request you to allow us to use the tape recorder just to ensure the correctness of the information when writing the report.

Shall we proceed? Yes…….1 No……2

1. May you kindly introduce yourself (age, marital status, residency/ location, and occupation and the position at the village government leadership)

   In our discussion today, we are going to focus on the management of Chronic disease conditions such as HIV, diabetes and hypertension

Community understanding of Chronic Disease Conditions

2. What do you understand by the phrase ‘chronic disease condition’?
3. What are the main chronic conditions people are suffering from in your communities? *(Probe examples; HIV, Diabetes and hypertension).*
4. Based on your experience, what activities or practices in the community that put one at risk of acquiring of HIV, diabetes and hypertension?

For peer review only - http://bmjopen.bmj.com/site/about/guidelines.xhtml

5. In your communities, how do people seek health care for HIV, Diabetes and hypertension? Probe on *Self-medication, consulting health facilities, spiritual healers, traditional healers and reasons for such option (probe one after the other for HIV, diabetes and hypertension)*

Health care services provided for chronic disease conditions (HIV, DM, HT) at the health facilities

6. As leaders, how would you describe the quality of services for people with HIV, DM and HT provided in the nearby health facility? (*Probe for: what do you like/dislike about them? probe trust in existing services)*
   a. Probe on whether they meet the needs of the patients in terms of screening facilities, prescription, availability of medicine, prescribers' *continuity of care, medicines availability, perceptions of healthcare provider expertise*)
   b. What are the key challenges affecting effective management of chronic conditions in your community?
   c. How best can these challenges be addressed to ensure effective management of chronic diseases?

Perceptions on INTE-COMM

7. The Ministry of Health is implementing a study on the integration of health care services for DM/HT/HIV in some selected community posts. This implies that patients with either DM, HT, HIV or both will receive care in a for all the diseases at once from their communities.
   a. What do you know about it (integrated model delivery)? *(probe on whether they have heard from others or is the first time, what ideas come up in your mind as leaders (probe for expectations)?*
   b. What are your views/perceptions towards such integrated model? *(Probe of the willingness of the patients to start and continue seeking care in such integrated model, stigma, and policy issues, cultural factors, etc.)*
   c. What are the possible positives and negatives about integration *e.g. time spent at the facility, perceived quality of care, stigma, costs, follow up support etc.)*

Contextual factors

8. Based on your experience as a leader in your community, what are the foreseen factors that can enable proper implementation of a community based integrated care service model? *Probe for: political support, policy environment, cultural values, beliefs and norms, religious influence, technical factors, etc.*

For peer review only

9. What are the foreseen factors that can hinder the proper implementation of a communitybased integrated care service model? *Probe for: political support, policy environment, cultural values, beliefs and norms, religious influence, technical factors, etc.*
10. What can be done to ensure the effective implementation of a community-based integrated care service model in your community?
11. What role can community leaders play in ensuring effective implementation of a community-based integrated care service model in your community?
12. What are the factors that can hinder the acceptability of INTE-COMM services? *Probe for: political support, policy environment, cultural values, beliefs and norms, religious influence, technical factors, etc*
13. What can be done to ensure the acceptability of a community-based integrated care service model in your community?
14. What role can community leaders play in ensuring acceptability of a community-based integrated care service model in your community?

Sustainability and scalability of INTE-COMM

15. What factors can hinder the sustainable provision of community-based integrated care for HIV, diabetes, and hypertension? *Probe for: political support, policy environment, cultural values, beliefs and norms, religious influence, technical factors, etc*
16. How can community-based integrated care for HIV, diabetes, and hypertension services be sustainably provided in Uganda? *Probe for: what should be done and by who? Including the role of community leaders, etc.*
17. What factors can hinder the scaling up of community-based integrated care for HIV, diabetes, and hypertension? *Probe for: political support, policy environment, cultural values, beliefs and norms, religious influence, technical factors, etc*
18. How can a community based integrated care for HIV, diabetes and hypertension be scaled up to other areas in Uganda? *Probe for the role of different stakeholders*

Thank you for your participation.

For peer review only

Observation Checklist

Instructions:

Observe and document the following in detail

1. Arrival and departure times for both patients and healthcare providers
2. Clinical procedures-how many service points are available, which services are offered at which point, and by whom?
3. Interactions/conversations among patients in the waiting areas, and conversations between patients and health care providers (what issues are talked about)
4. Behaviours of patients-isolating themselves from the rest, patients body language, hunger, restlessness, etc.
5. Behaviours of healthcare providers-language and tone used to communicate with patients, body language/facial expressions, time management, prioritizing patients against other activities, etc.)
6. Sitting arrangements of patients (congestion, comfortable seats, enough seats, exposure to difficult weather conditions, etc.
7. Location of the service points (if offers privacy and confidentiality)
   If health information/education sessions are offered, by whom, what content, participation/attentiveness of patients/asking questions and receiving responses, etc.

For peer review only
